# Efficient Low‐temperature Ammonia Cracking Enabled by Strained Heterostructure Interfaces on Ru‐free Catalyst

**DOI:** 10.1002/adma.202502034

**Published:** 2025-04-28

**Authors:** Pei Xiong, Jiangtong Li, Zhihang Xu, Yashan Lin, Robert David Bennett, Yi Zhang, Wei‐Min Tu, Ye Zhu, Yun‐Liang Soo, Tai‐Sing Wu, Molly Meng‐Jung Li

**Affiliations:** ^1^ Department of Applied Physics The Hong Kong Polytechnic University Hong Kong 999077 China; ^2^ The Institute for Advanced Studies Wuhan University Wuhan 430072 China; ^3^ CSIRO Energy, Clayton Laboratories Clayton South VIC 3168 Australia; ^4^ Department of Physics National Tsing Hua University Hsinchu 30013 Taiwan; ^5^ Research Institute of Smart Energy (RISE) The Hong Kong Polytechnic University Hong Kong 999077 China; ^6^ National Synchrotron Radiation Research Center Hsinchu 30076 Taiwan

**Keywords:** ammonia cracking, core@shell catalysts, dynamic strain evolution, heterostructure interface, lattice strain

## Abstract

Ammonia (NH_3_) has emerged as a promising liquid carrier for hydrogen (H_2_) storage. However, its widespread adoption in H_2_ technology is impeded by the reliance on costly Ru catalysts for low‐temperature NH_3_ cracking reaction. Here, a strained heterostructure Co@BaAl_2_O_4−x_ core@shell catalyst is reported that demonstrates catalytic performance at low reaction temperatures comparable to most Ru‐based catalysts. This catalyst exhibits exceptional activity across a range of space velocity conditions, maintaining high conversion rates at 475 to 575 °C and achieving an impressive H_2_ production rate of 64.6 mmol H_2_ g_cat_^−1^ min^−1^. Synchrotron X‐ray absorption spectroscopy, synchrotron X‐ray diffraction, and kinetic studies are carried out to elucidate the dynamic changes of the strained heterostructure interface of Co‐core and BaAl_2_O_4−x_‐overlayer under catalytic working conditions. The performance enhancement mechanisms are attributed to the tensile strained Co surface encapsulated in the defective BaAl_2_O_4−x_, which enhances NH_3_ adsorption and facilitates the rate‐determining N─H dissociation. Furthermore, the strain release and restoration during NH_3_ dehydrogenation enable efficient nitrogen desorption, preventing active site poisoning. This work highlights the effectiveness of lattice strain engineering and the development of synergistic strong metal‐support interfaces between active metal nanoparticles and oxide support to boost low‐temperature NH_3_ cracking.

## Graphical abstract

Leveraging the strong metal‐support interaction effect, heterostructured interfaces in a Co‐based catalyst are designed to enable low‐temperature ammonia (NH_3_) decomposition. The induced tensile lattice strain within the core‐shell structure modulates *d*‐band center of Co, enhancing NH_3_ adsorption and facilitating N─H bond dissociation. Furthermore, the dynamic lattice strain release and restoration mechanism promotes NH_3_ dehydrogenation and by‐product desorption, effectively optimizing the reaction pathway and ensuring sustained catalytic activity.

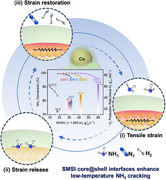

## 1 Introduction

Hydrogen (H_2_) has received considerable attention as a clean energy source because, in fuel cells, it reacts with oxygen to generate electricity, producing only water as a by‐product and achieving higher energy conversion efficiencies compared to traditional fossil fuel‐based systems.^[^ ^1^ ^]^ However, achieving the “H_2_ economy” requires safe and cost‐effective H_2_ storage.^[^ ^2^ ^]^ In this regard, ammonia (NH_3_) is considered a promising H_2_ carrier due to its i) well‐established production and distribution infrastructure, ii) high volumetric and gravimetric H_2_ density, and iii) ability to decompose into CO_x_‐free H_2_.^[^ ^3^ ^]^

For energy applications, low‐temperature NH_3_ decomposition is preferred. Nevertheless, the most efficient low‐temperature NH_3_ decomposition catalytic systems rely heavily on ruthenium (Ru)‐based catalysts,^[^ ^3^, ^4^ ^]^ which are costly and limit large‐scale implementation.^[^ ^5^ ^]^ As a result, research has shifted toward alternative catalysts using more abundant non‐noble metals such as iron (Fe),^[^ ^6^ ^]^ cobalt (Co),^[^ ^5^ ^]^ or nickel (Ni).^[^ ^7^ ^]^ Among these, Co stands out due to its nitrogen binding energy being close to the optimal range of Ru and its lower susceptibility to poisoning.^[^ ^8^ ^]^ Therefore, Co‐based catalysts have emerged as promising candidates for NH_3_ decomposition.^[^ ^5^, ^9^ ^]^

However, the low‐temperature kinetics of NH_3_ decomposition are primarily hindered by inefficient NH_3_ adsorption and N─H bond dissociation on Co‐based catalysts.^[^ ^5^ ^]^ While most Co‐based catalysts demonstrate satisfactory performance only at high temperatures (>600 °C), significant efforts have been directed toward achieving complete hydrogen generation at lower temperatures (<500 °C).^[^ ^10^ ^]^ Consequently, developing more efficient Co‐based catalysts capable of enhancing NH_3_ adsorption and facilitating N─H bond dissociation at lower temperatures remains a critical challenge. Innovative catalyst material design strategies are urgently needed to achieve efficient and cost‐effective H_2_ production from NH_3_ decomposition.

Lattice strain engineering has emerged as an effective strategy to enhance catalytic performance by modulating the interactions between active sites and reactants.^[^ ^11^ ^]^ This approach leverages lattice strain to influence the electronic structure of catalysts, thereby tuning their surface adsorption properties. For example, Jin et al. demonstrated that continuously adjusting the lattice strain of Pt(100) within a range of ‐5.1% to +5.9% effectively modulated the adsorption strength of reaction intermediates involved in methanol oxidation and H_2_ evolution reactions.^[^ ^12^ ^]^ Similarly, Fan et al. investigated the impact of tensile strain on the adsorption energy of catalysts for nitrate reduction reaction, revealing that introducing tensile strain into Ti_2_CO_2_ MXene shifted the *d*‐band center closer to the Fermi level, thereby improving nitrogen (N_2_) adsorption and activation.^[^ ^13^ ^]^ These findings align with the Sabatier principle, which emphasizes achieving optimal adsorption strength to maximize catalytic efficiency. However, the lattice strain strategy has not yet been explored in thermocatalytic NH_3_ cracking.

Building on this concept, introducing lattice strain into Co‐based NH_3_ decomposition catalysts offers a promising approach for further tuning and optimizing catalytic performance. Nonetheless, lattice strain is not static and may dynamically evolve under reaction conditions, presenting additional challenges in understanding its role in catalytic performance. Real‐time analysis of lattice strain evolution during catalytic reactions is therefore essential for elucidating its regulatory effects on reaction pathways, enabled by advancements in in situ and *operando* characterization techniques. Such insights will provide a foundation for designing and optimizing advanced Ru‐free catalysts with improved efficiency for NH_3_ decomposition.

Herein, we leverage our recently established core@shell catalyst synthesis strategy to fabricate Co@BaAl_2_O_4−x_ catalysts with a distinct tensile strain at the heterostructure interface, induced by the strong metal‐support interaction (SMSI) effect. This catalyst exhibits exceptional activity for NH_3_ decomposition, achieving nearly complete NH_3_ conversion at a moderate temperature of 475 to 575 °C and maintaining high efficiency under high flow rate conditions. Comprehensive kinetic analysis and in situ characterizations unveil the critical role of the strained Co and BaAl_2_O_4−x_ heterostructure interface in enhancing catalytic performance. The tensile strain at the interface elevates the *d*‐band center of Co, thereby strengthening NH_3_ adsorption and facilitating N─H bond dissociation through modulating electronic structures. Furthermore, the subsequent release of tensile strain after NH_3_ adsorption/activation promotes efficient desorption of reaction products, preventing active site poisoning and ensuring sustained catalytic activity. The elucidation of this unique dynamic strain evolution mechanism at the heterostructure interface provides valuable insights into the influence of lattice strain on catalytic processes. These findings highlight the potential of heterostructure interface engineering to advance NH_3_ decomposition catalysis and contribute to the broader development of sustainable and efficient catalyst design strategies in clean energy applications.

## 2 Results and Discussion

### 2.1 SMSI Core@Shell Catalyst Composition Optimization for NH_3_ Decomposition Reaction

**Figure 1:**
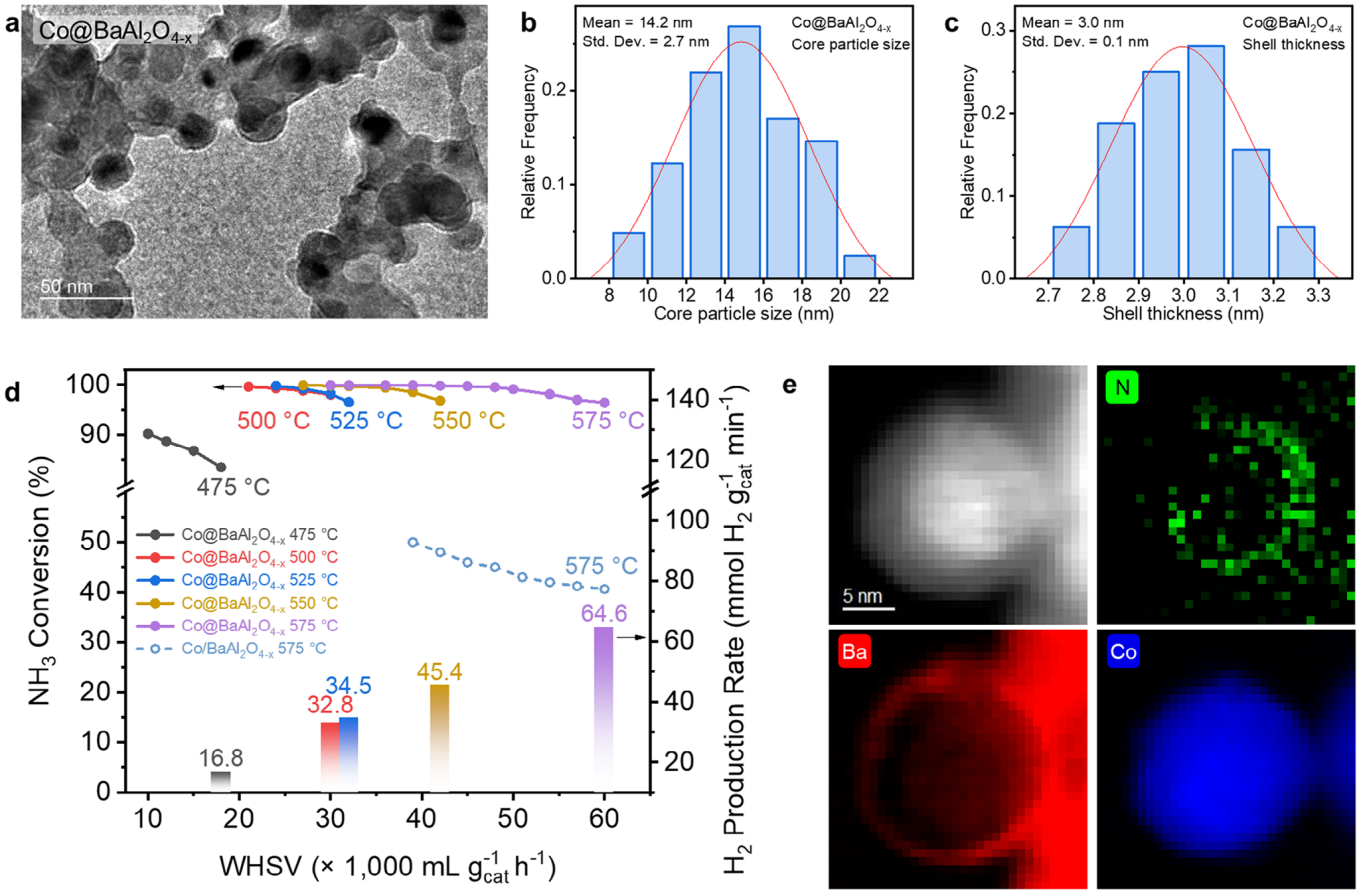
SMSI Core@shell catalyst composition optimization for NH_3_ decomposition reaction. a) TEM image of Co@BaAl_2_O_4−x_ heterostructure. b) Core particle size distribution of Co@BaAl_2_O_4−x_ heterostructure measured from TEM images. c) Shell‐thickness distribution of Co@BaAl_2_O_4−x_ heterostructure measured from TEM images. d) NH_3_ conversion (solid lines) and corresponding H_2_ production rate (bar) as a function of WHSV over Co@BaAl_2_O_4−x_, as well as Co/BaAl_2_O_4−x_ at 575 °C for comparison (dashed line). e) Scanning transmission electron microscopy (STEM) images and electron energy loss spectroscopy (EELS) element distribution maps of post‐reaction Co@BaAl_2_O_4−x_.

The SMSI effect observed in encapsulated heterostructured catalysts is often associated with outstanding performance. Using our recently developed Tammann temperature‐guided SMSI core@shell synthesis strategy,^[^ ^14^ ^]^ we have synthesized various catalysts with different active metals and promoters, optimizing their performance for NH_3_ decomposition. Among these, Co‐Ba‐Al‐O composition exhibits superior activity for the low temperature (<600 °C) range (Figure S1, Supporting Information).^[^ ^14^ ^]^ Further refinement of the Co‐Ba‐Al‐O catalyst was achieved by adjusting synthesis parameters, including the Ba/Al molar ratio, Co content, calcination temperature, and reduction temperature (Figure S2, Supporting Information). The optimal catalyst was synthesized with a Ba/Al molar ratio of 1/2, ca. 31.6 wt.% Co content, a calcination temperature of 500 °C, and a reduction temperature of 700 °C. Transmission electron microscopy (TEM, **Figure** **1**a) characterizations reveals a core@shell heterostructure with an average Co particle size of 14.2 ± 2.7 nm (Figure 1b) and a shell thickness of 3.0 ± 0.1 nm (Figure 1c). See Figure S3 (Supporting Information) for size analysis details. X‐ray photoelectron spectroscopy (XPS) analysis confirms the presence of oxygen vacancies in the BaAl_2_O_4_ shell due to its formation under reducing conditions (Figure S4a, Supporting Information),^[^ ^14^ ^]^ thereby the sample is denoted as Co@BaAl_2_O_4−x_. When evaluated for NH_3_ decomposition across various temperatures and weight hourly space velocities (WHSVs), Co@BaAl_2_O_4−x_ demonstrates exceptional performance, achieving an impressive H_2_ production rate of 64.6 mmol H_2_ g_cat_^−1^ min^−1^ at a high WHSV of 60000 mL_NH3_ g_cat_^−1^ h^−1^ (Figure 1d). Notably, the catalyst maintains nearly complete NH_3_ conversions between 475 °C and 575 °C, rivalling the performance of most Ru‐based catalysts, which typically required 450 to 550 °C to achieve ∼99% NH_3_ conversion.^[^ ^15^ ^]^

Intriguingly, the scanning transmission electron microscope coupled with electron energy loss spectroscopy (STEM‐EELS) analysis of the post‐reaction Co@BaAl_2_O_4−x_ catalyst reveals a pronounced accumulation of nitrogen species specifically at the core@shell interface (Figure 1e). This observation strongly suggests that NH_3_ decomposition predominantly occurs at the interface between the Co core and the BaAl_2_O_4−x_ shell, with the oxygen vacancies in the shell ensuring permeability for reactants and facilitating the catalytic process (Figures S5 and S6, Supporting Information). The distinctive core@shell structure creates the unique heterostructure interface between metal Co and BaAl_2_O_4−x_ support (will be discussed in the following), which suggests a critical role of the metal support interactions in enhancing the catalytic performance.

### 2.2 Improved Reaction Kinetics at the Core@Shell Heterostructure Interface

A series of reaction kinetic studies were conducted to uncover the enhancement mechanism of the Co@BaAl_2_O_4−x_ core@shell heterostructure. For comparison, a sample with the same composition but without a core@shell structure, denoted as Co/BaAl_2_O_4−x_, was synthesized. Both samples were prepared with a similar size distribution of Co nanoparticles to minimize size effects on catalytic performance (Figure S5e,f; Table S1, Supporting Information).

The catalytic activities of the samples were evaluated for NH_3_ decomposition over a temperature range of 200 °C to 650 °C at a WHSV of 30 000 mL_NH3_ g_cat_^−1^ h^−1^. The core@shell Co@BaAl_2_O_4−x_ catalyst exhibits superior NH_3_ conversion compared to the supported Co/BaAl_2_O_4−x_ catalyst (Figure S5g, Supporting Information), highlighting the impact of its core@shell heterostructure. The onset temperature for NH_3_ decomposition is 200 °C for Co@BaAl_2_O_4−x_, compared to 250 °C for Co/BaAl_2_O_4−x_. With rising temperature, both catalysts show increasing NH_3_ conversion, consistent with the endothermic nature of the reaction. Notably, Co@BaAl_2_O_4−x_ achieves near‐complete NH_3_ conversion at 500 °C, whereas Co/BaAl_2_O_4−x_ requires 550 °C to reach thermodynamic equilibrium under identical conditions. Furthermore, the non‐core‐shell Co/BaAl_2_O_4−x_ catalyst displays pronounced susceptibility to high WHSV conditions, with NH_3_ conversion decreasing sharply as WHSV increased (Figure 1d), contrasting with the robust performance of the core@shell counterpart.

**Figure 2:**
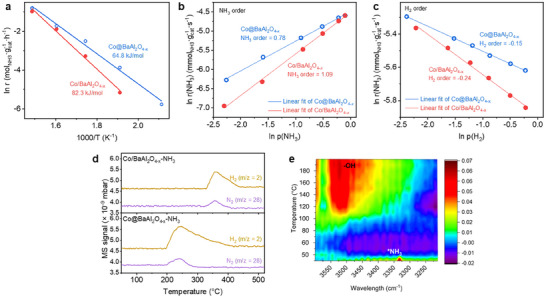
Improved reaction kinetics in the core@shell heterostructure interface. a) *E*_a_ determined for the NH_3_ decomposition reaction on the Co@BaAl_2_O_4−x_ and Co/BaAl_2_O_4−x_. b) Reaction orders of NH_3_ determined for the NH_3_ decomposition reaction at 350 °C on the Co@BaAl_2_O_4−x_ and Co/BaAl_2_O_4−x_. c) Reaction orders of H_2_ determined for the NH_3_ decomposition reaction at 350 °C on the Co@BaAl_2_O_4−x_ and Co/BaAl_2_O_4−x_. d) NH_3_‐TPSR over the Co@BaAl_2_O_4−x_ and Co/BaAl_2_O_4−x_. e) Changes in intensity of peaks at 3400–3500 cm^−1^ (‐OH species formed at the heterostructured interface) and 3320–3340 cm^−1^ (adsorbed *NH_3_ species on Co surface) of in situ diffuse reflectance infrared Fourier transform spectroscopy (DRIFTS experiments with NH_3_ adsorption of Co@BaAl_2_O_4−x_ from room temperature to 200 °C.

Arrhenius plots (**Figure** **2**a), generated by linearly fitting ln(reaction rate) versus 1/T in the 200–400 °C range (low‐conversion regime), reveal distinct apparent activation energies (*E*_a_) for the two catalysts. Co@BaAl_2_O_4−x_ exhibits a lower *E*_a_ of 64.8 kJ mol^−1^, comparable to the reported low *E*_a_ values for the Ru‐based catalysts (ranging from 50 to 130 kJ mol^−1^),^[^ ^10^, ^16^ ^]^ while Co/BaAl_2_O_4−x_ shows a much higher *E*_a_ of 82.3 kJ mol^−1^. This reduction in *E*_a_ underscores the role of the core@shell heterostructure in facilitating NH_3_ decomposition.

Reaction order studies at 350 °C with varying NH_3_, H_2_, and N_2_ concentrations have provided further insights into the catalytic process (see experimental details in the Supporting Information; the results are presented in Figure 2b,c; Figure S7, Supporting Information). As depicted in Figure 2b, the core@shell configuration reduces the positive reaction order for NH_3_ from +1.09 (Co/BaAl_2_O_4−x_) to +0.78 (Co@BaAl_2_O_4−x_). The lower NH_3_ order of Co@BaAl_2_O_4−x_ indicates that the NH_3_ reaction rate becomes less sensitive to changes in NH_3_ concentration. This phenomenon typically occurs when the active sites on the catalyst surface are highly occupied or saturated by NH_3_ molecules, suggesting that the structure enhances NH_3_ adsorption on active Co sites by alleviating adsorption limitations typically observed in conventional Co catalysts.^[^ ^3^, ^5^ ^]^ Additionally, as illustrated in Figure 2c, Co@BaAl_2_O_4−x_ displays a less negative H_2_ reaction order (−0.15 vs −0.24 for Co/BaAl_2_O_4−x_), indicating more efficient hydrogenH removal and reduced hydrogen poisoning during NH_3_ dehydrogenation.^[^ ^3^, ^17^ ^]^

NH_3_ temperature‐programmed surface reaction (NH_3_‐TPSR) analysis supports the kinetic study findings. After pre‐adsorbing NH_3_ at 50 °C for 60 min and purging with argon (Ar) (leaving only adsorbed NH_3_ on the catalyst surface), temperature ramping reveals higher H_2_ and N_2_ signal intensities for Co@BaAl_2_O_4−x_ compared to Co/BaAl_2_O_4−x_ (Figure 2d), confirming core@shell enhances NH_3_ adsorption. On the other hand, the in situ diffuse reflectance infrared Fourier transform spectroscopy (DRIFTS) reveals notable ‐OH peaks during temperature increases, coinciding with the disappearance of adsorbed NH_3_ species (see Figure 2e).^[^ ^3a^ ^]^ These observations indicate N─H bond cleavage during NH_3_ dehydrogenation at the heterostructure interface. The H^+^ ions from NH_x_ dehydrogenation on the Co surface bind to BaAl_2_O_4−x_ oxygen sites, forming ‐OH groups.^[^ ^2^ ^]^

The above results have demonstrated that the unique SMSI core@shell heterostructure in Co@BaAl_2_O_4−x_ enhances key steps in NH_3_ decomposition by reducing activation energy, improving NH_3_ adsorption, facilitating hydrogen removal, and promoting N─H bond cleavage. This highlights its potential as an efficient catalyst configuration for NH_3_ decomposition reactions.

### 2.3 Identifying Lattice Strain at the Core@Shell Heterostructure Interface

**Figure 3:**
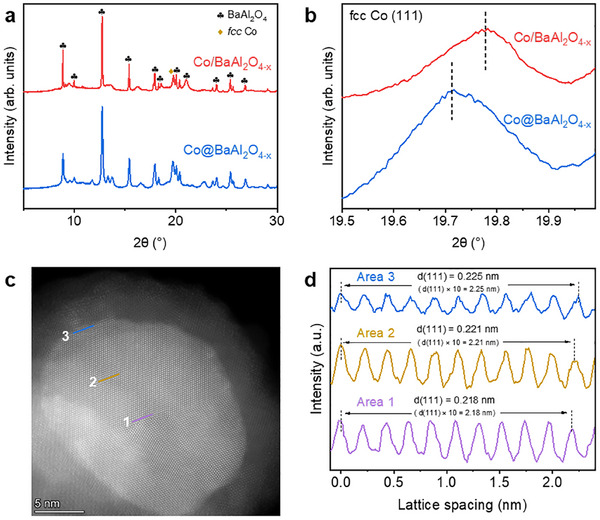
Identifying lattice strain at the core@shell heterostructure interface. a) SXRD patterns of Co@BaAl_2_O_4−x_ and Co/BaAl_2_O_4−x_ (λ  =   0.7000 Å). b) Enlarged views of diffraction peaks of *fcc* Co(111) crystal facet in Co@BaAl_2_O_4−x_ and Co/BaAl_2_O_4−x_ in Figure 3a. c) Spherical aberration‐corrected scanning transmission electron microscopy (AC‐STEM) image of the Co@BaAl_2_O_4−x_. d) Processed lattice fringe images of areas 1, 2, and 3 in Figure 3c, respectively.

The pronounced enhancement in the catalytic performance of the core@shell heterostructure catalyst has prompted further investigations into its unique structural and electronic properties. **Figure** **3**a presents the synchrotron X‐ray diffraction (SXRD) patterns of Co@BaAl_2_O_4−x_ and Co/BaAl_2_O_4−x_, where both samples exhibit distinct crystalline phases of Co and BaAl_2_O_4_. However, a closer examination of the Co(111) diffraction peak reveals notable differences. As shown in Figure 3b, the Co(111) peak in Co@BaAl_2_O_4−x_ shifts toward lower angles compared to Co/BaAl_2_O_4−x_. This shift indicates an expansion of the Co lattice in the core@shell samples. The refined lattice parameters derived from SXRD, detailed in Table S1 (Supporting Information), confirm this observation by showing that the Co cell volume of Co@BaAl_2_O_4−x_ is larger than that of Co/BaAl_2_O_4−x_.

To further investigate the lattice characteristics at the Co@BaAl_2_O_4−x_ heterostructure interface, AC‐STEM was conducted. Figure 3c highlights a selected core@shell particle of Co@BaAl_2_O_4−x_, where lattice spacings are measured across three regions extending outward from the core center: area 1 (purple line), area 2 (brown line), and area 3 (blue line). The measured lattice spacings of Co(111) in these regions are 2.18, 2.21, and 2.25 Å, respectively (Figure 3d). The notable increase in lattice spacing observed in area 3 corresponds to a tensile strain of ≈+3.2% relative to the inner core. This direct visualization confirms lattice expansion at the core@shell heterostructure interface.

**Figure 4:**
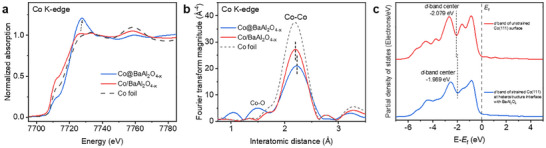
Identifying the electronic structure of Co at the core@shell heterostructure interface. a) Co K‐edge XANES spectra of as‐synthesized Co@BaAl_2_O_4−x_ and Co/BaAl_2_O_4−x_, as well as Co foil reference. b) Co K‐edge Fourier transformed EXAFS spectra of as‐synthesized Co@BaAl_2_O_4−x_ and Co/BaAl_2_O_4−x_, as well as Co foil reference. c) Projected *d*‐band partial density of states of Co atoms in strained Co(111) at the heterostructure interface with BaAl_2_O_4_ and unstrained Co(111) surface.

X‐ray absorption spectroscopy (XAS) was conducted to probe the electronic structure of Co in Co@BaAl_2_O_4−x_ and Co/BaAl_2_O_4−x_ before the reaction. Co K‐edge X‐ray absorption near‐edge structure (XANES) analyses reveal that Co@BaAl_2_O_4−x_ exhibits a higher absorption edge position and greater white‐line intensity compared to Co/BaAl_2_O_4−x_ (**Figure** **4**a). Similarly, XPS analysis shows an increase in binding energy for Co 3p (Figure S8a, Supporting Information). Further insights are provided by extended X‐ray absorption fine structure (EXAFS) spectra, which shows that Co@BaAl_2_O_4−x_ has a broader Co─Co peak centered at a longer bond distance compared to Co/BaAl_2_O_4−x_ and metallic Co foil. EXAFS fitting results have confirmed the slightly elongated Co─Co bonds in Co@BaAl_2_O_4−x_ (Figure 4b; Figure S9; Table S2, Supporting Information). The comprehensive structural and electronic characterizations indicate that tensile strain at the core@shell heterostructure interface induces a higher valence state for Co within the encapsulated configuration.

The observed variation in the valence state of Co can be attributed to the well‐established strain‐induced *d*‐band modification (Figure S10, Supporting Information).^[^ ^18^ ^]^ The tensile strain experienced by Co in the core@shell heterostructure leads to a reduction in electron density and diminished *d*‐band overlap between neighboring Co atoms.^[^ ^19^ ^]^ These electronic changes, manifested by the higher valence state observed in XAS analysis, result in a narrowing of the *d*‐band and a positive shift of its centre to maintain the degree of *d*‐band filling.^[^ ^20^ ^]^ This shift enhances Co's adsorption capacity for the NH_3_ molecule,^[^ ^21^ ^]^ thereby significantly improving the NH_3_ decomposition performance of the Co@BaAl_2_O_4−x_ catalyst.

To further validate these electronic effects, density functional theory (DFT) simulations were performed. Theoretical models compared the strained Co(111) at the heterostructure interface with BaAl_2_O_4_ to the unstrained Co(111) surface. The partial density of states analysis reveals that the *d*‐band center for the strained Co(111) shifts upward by +0.304 eV, bringing it closer to the Fermi level compared to the unstrained surface (Figure 4c). This upward shift aligns with our experimental observations and supports the concept that tensile strain enhances Co's reactivity by facilitating NH_3_ adsorption and activation. Details on the construction of DFT models and electronic structure analysis are provided in the Supplementary Information (Figures S11 and S12, Supporting Information). This theoretical evidence complements the experimental results from kinetic studies, providing a critical understanding of the strain‐enhanced catalytic performance.

### 2.4 Dynamic Lattice Strain Release and Restoration in the NH_3_ Decomposition Reaction Cycle

The aforementioned findings have provided evidence of the critical role of tensile strain at the core@shell heterostructure interface in enhancing reactant adsorption by modulating electronic structures. To further understand how strain evolves during the NH_3_ decomposition reaction, a systematic investigation was conducted using in situ characterization techniques to monitor changes at each reaction step.

**Figure 5:**
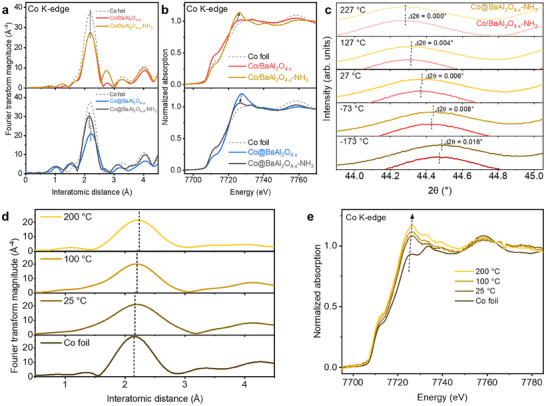
Dynamic lattice strain release and restoration in the NH_3_ decomposition reaction cycle. a) Upper panel: Co K‐edge Fourier transformed EXAFS spectra of Co/BaAl_2_O_4−x_ and Co/BaAl_2_O_4−x_‐NH_3_, as well as Co foil reference; Lower panel: Co K‐edge Fourier transformed FT‐EXAFS spectra of Co@BaAl_2_O_4−x_ and Co@BaAl_2_O_4−x_‐NH_3_, as well as Co foil reference. b) Upper panel: Co K‐edge XANES spectra of Co/BaAl_2_O_4−x_ and Co/BaAl_2_O_4−x_‐NH_3_, as well as Co foil reference; Lower panel: Co K‐edge XANES spectra of Co@BaAl_2_O_4−x_ and Co@BaAl_2_O_4−x_‐NH_3_, as well as Co foil reference. c) Enlarged views of diffraction peaks of *fcc* Co(111) crystal facet in in situ SXRD patterns of Co@BaAl_2_O_4−x_‐NH_3_ and Co/BaAl_2_O_4−x_‐NH_3_ with temperature raising from −173 to 227 °C (Full patterns are shown in Figure S14, Supporting Information). d) In situ Co K‐edge Fourier transformed FT‐EXAFS of Co@BaAl_2_O_4−x_‐NH_3_ under heating from 25 to 200 °C, as well as Co foil reference. e) In situ Co K‐edge X‐ray absorption near‐edge structure (XANES) spectra of Co@BaAl_2_O_4−x_‐NH_3_ under heating at different temperatures, as well as Co foil reference.

Co@BaAl_2_O_4−x_ and Co/BaAl_2_O_4−x_ samples with pre‐adsorbed NH_3_ were prepared, denoted as Co@BaAl_2_O_4−x_‐NH_3_ and Co/BaAl_2_O_4−x_‐NH_3_, respectively. XAS analysis reveals that NH_3_ adsorption causes the Co─Co peak in the Co K‐edge EXAFS of Co@BaAl_2_O_4−x_‐NH_3_ to become narrower and more symmetrical (close to that of Co foil). This contrasts with the broader, asymmetrical peak observed in the strained Co@BaAl_2_O_4−x_ (**Figure** **5**a; Figure S13; Table S3, Supporting Information). In comparison, no significant changes in Co─Co bond lengths are observed for Co/BaAl_2_O_4−x_ upon NH_3_ adsorption. Additionally, XANES analysis shows that Co@BaAl_2_O_4−x_‐NH_3_ exhibits a lower absorption edge position and reduced white‐line intensity compared to strained Co@BaAl_2_O_4−x_ (Figure 5b), indicating strain release and a reduced oxidation state upon NH_3_ adsorption.

Interestingly, while literature reports suggest that NH_3_ adsorption on transition metal surfaces typically increases the metal's valence state due to interactions with nitrogen species,^[^ ^22^ ^]^ this trend is observed only for Co/BaAl_2_O_4−x_‐NH_3_. In contrast, the opposite behavior is noted for Co@BaAl_2_O_4−x_‐NH_3_, where strain release induced significant electronic changes. This strain release phenomenon brings adjacent Co atoms closer together, increasing *d*‐orbital overlap and causing a downward shift in the *d*‐band center. As a result, adsorption strength on the strain‐released surface is reduced, which can facilitate the desorption of reaction products.

To demonstrate dynamic strain evolution during the NH_3_ decomposition cycle, specifically, strain restoration after product desorption, in situ heating experiments were performed on Co@BaAl_2_O_4−x_‐NH_3_. SXRD monitoring of the Co(111) peak during heating reveals that as pre‐adsorbed NH_3_ molecules desorb from the surface, the Co(111) peak shifts back toward lower angles (Figure 5c; Figure S14, Supporting Information). At 227 °C (around the H_2_ evolution temperature in NH_3_‐TPSR; Figure 2d), the peak aligns with that of strained Co(111) in clean Co@BaAl_2_O_4−x_ at higher temperatures. Complementary in situ XAS measurements shows a gradual increase in Co─Co bond length and valence state during heating, confirming strain restoration as desorption completed (Figure 5d,e; Figure S15; Table S4, Supporting Information).

**Figure 6:**
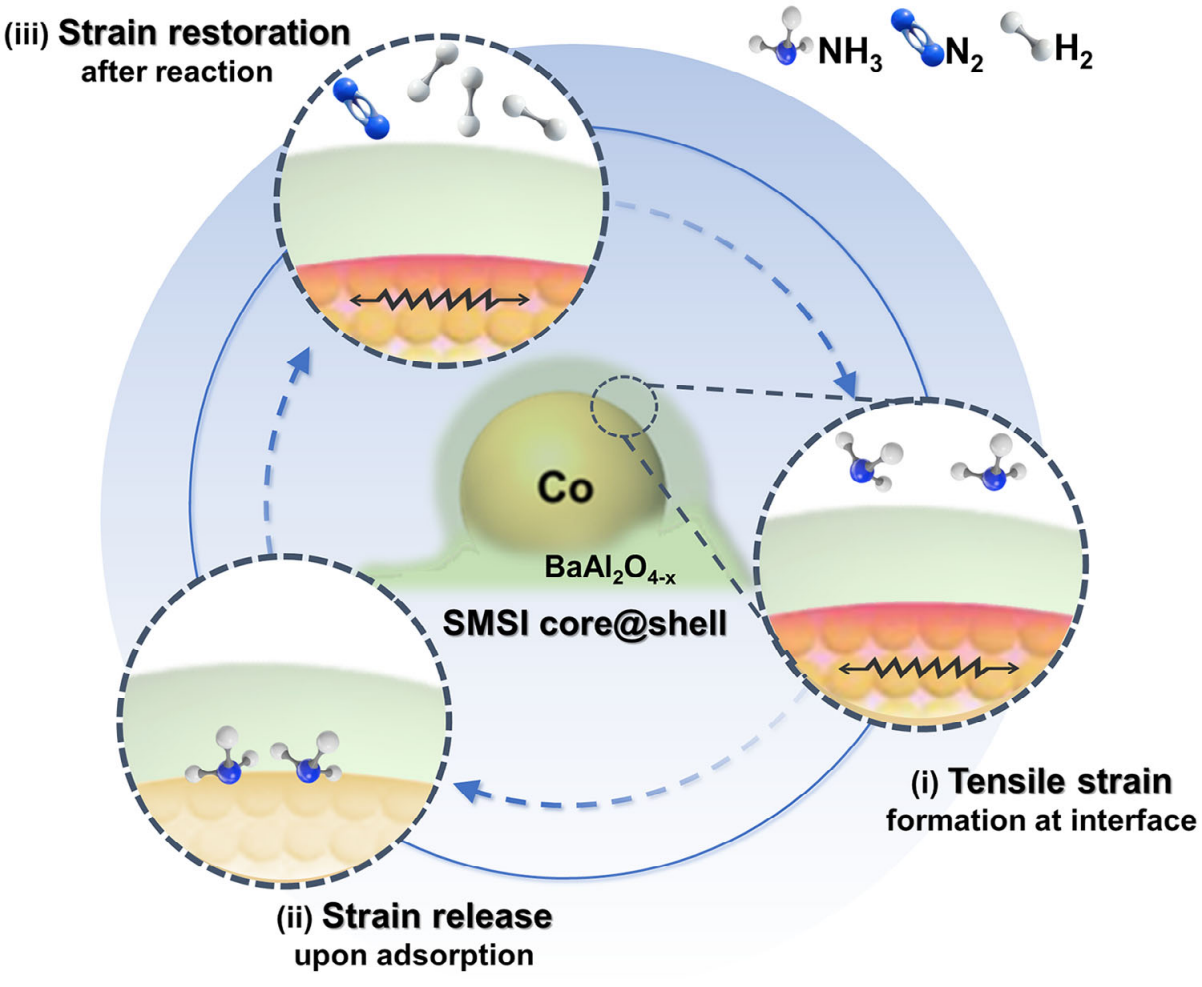
Schematic of the dynamic lattice strain evolution mechanism for NH_3_ decomposition over core@shell heterostructured Co@BaAl_2_O_4−x_ catalyst.

The dynamic lattice strain evolution mechanism for NH_3_ decomposition over the core@shell heterostructured Co@BaAl_2_O_4−x_ catalyst is summarized as follows and illustrated in **Figure** **6**.

i) Tensile strain formation: At the core@shell heterostructure interface, tensile strain forms and elevates the *d*‐band center of Co, enhancing NH_3_ adsorption and facilitating its reaction. ii) Strain released upon adsorption: Strain is released when NH_3_ adsorbs and reacts on the surface. This strain release shifts the *d*‐band downward, reducing adsorption strength and promoting product desorption. iii) Strain restoration after reaction: Following product desorption (e.g., N_2_ or H_2_), tensile strain at interfacial sites is restored, preparing the catalyst for subsequent reaction cycles.

Such dynamic strain evolution mechanism discovered in this research provides a comprehensive understanding of the interplay between lattice strain, NH_3_ adsorption, N─H dissociation, and strain restoration in the core@shell heterostructured Co@BaAl_2_O_4−x_ catalyst system. These new mechanistic insights also highlight the importance and effectiveness of strain engineering and interface modulation for enhancing NH_3_ decomposition catalysis.

## 3 Conclusion

In conclusion, this study highlights the dynamic lattice strain evolution mechanism at the interface of core@shell heterostructures during NH_3_ decomposition. Employing a core@shell catalyst synthesis approach driven by the SMSI effect, we successfully engineered heterostructured interfaces within the Ru‐free catalyst formulation, Co@BaAl_2_O_4−x_, which operates efficiently under low‐temperature reaction conditions. Our findings reveal that the induced lattice strain at the Co core and BaAl_2_O_4−x_ overlayer interface modulates the *d*‐band center of Co, thereby altering its electronic structure to enhance NH_3_ adsorption and facilitate N─H bond dissociation. Furthermore, the release of tensile strain during NH_3_ adsorption and activation promotes efficient desorption of reaction products, preventing active site poisoning and ensuring sustained catalytic performance. This dynamic lattice strain evolution mechanism not only facilitates the reaction pathway but also represents a significant advancement in renewable catalysis research, offering promising application opportunities in energy and environmental sustainability.

## 4 Experimental Section

### Preparation Methods for Optimal Co@BaAl_2_O_4−x_ and Comparative Co/BaAl_2_O_4−x_

The core@shell Co@BaAl_2_O_4−x_ catalyst was fabricated through a co‐precipitation method with automated pH control, followed by a thermal treatment process. The typical synthesis procedure began by preparing a 50 mL solution containing 0.1 M Co(NO_3_)_2_, 0.05 M Ba(NO_3_)_2_, and 0.1 m Al(NO_3_)_3_ dissolved in deionized (DI) water, maintaining a molar ratio of 2:1:2. At ambient temperature, this mixed‐metal solution was added dropwise into a stirred reactor (capacity ranging from 500 mL to 2 L) containing 0.5 m Na_2_CO_3_ solution. The addition rate of the metal solution was controlled using a syringe pump, with a flow rate typically set between 0.1 and 2.0 mL min⁻¹. To ensure homogeneity, the mixture was stirred vigorously throughout the process. Simultaneously, the pH of the reaction solution was kept stable (pH = 12.5) by gradually introducing 4.0 m NaOH solution via another syringe pump. After all the metal nitrate solutions were completely added, the suspension was aged for 16 h. The resulting mixture was then filtered and rinsed with DI water until the effluent reached a neutral pH (≈7.0). The collected wet solid was re‐dispersed in 200 mL of acetone and stirred at room temperature for 2 h. The resulting solid was vacuum filtered, washed with acetone, and dried overnight in a vacuum oven at room temperature. The dried material was calcined in air at 500 °C for 4 h and subsequently subjected to thermal treatment in a H_2_/Ar (5/95, v/v) atmosphere at 700 °C for 2.5 h, which facilitated the formation of the core@shell Co@BaAl_2_O_4−x_ catalyst with Co loading of ca. 31.6 wt.%.

In comparison, the Co/BaAl_2_O_4−x_ catalyst was synthesized using a wet impregnation method. The synthesis procedure is described as follows: First, the BaAl_2_O_4−x_ support was prepared by calcining a mixture of Ba(NO_3_)_2_ and Al_2_O_3_ directly in a H_2_/N_2_ (5/95, v/v) atmosphere at 700 °C for 2.5 h. This resulting BaAl_2_O_4−x_ support was then combined with an appropriate amount of Co(NO_3_)_2_ in 50 mL of ethanol. The Co content in the Co/BaAl_2_O_4−x_ catalyst was adjusted to ≈32.9 wt.%, matching the composition of the Co@BaAl_2_O_4−x_ catalyst. The resulting mixture was continuously stirred at 800 rpm for 12 h on a hot plate magnetic stirrer, maintaining a temperature of 50 °C. Following the stirring process, the mixture was transferred to a vacuum oven set at 50 °C for 8 h to remove the ethanol. The dried sample was then calcined in air at 500 °C for 4 h, followed by thermal treatment in a H_2_/Ar (5/95, v/v) atmosphere at 700 °C for 2.5 h, ultimately producing the Co/BaAl_2_O_4−x_ catalyst.

### Material Characterizations

The phases and crystallographic structures of the samples were analyzed using SXRD, conducted on the Powder Diffraction (PD) beamline at the Australian Synchrotron (AS). The measurements were performed with a photon energy of 17.711 keV (wavelength λ = 0.7000 Å). To investigate the microstructure and phase composition, both STEM in high‐angle annular dark field (HAADF) mode and transmission electron microscopy (TEM) were utilized. The analyses were carried out using a double‐Cs‐corrected STEM (Spectra 300, TFS, USA) and a STEM/TEM instrument (JEM‐2100F, JEOL, Japan) integrated with a Gatan Enfina electron spectrometer (USA). X‐ray absorption fine structure (XAFS) spectra were collected in fluorescence mode at beamline BL01C of the Taiwan Light Source at the National Synchrotron Radiation Research Center (NSRRC). Photon energy scanning was performed using a Si(111) double crystal monochromator (DCM). In situ XAFS were measured in fluorescence mode using a 19SSD detector at the beamline BL01B1 of the SPring‐8 of Japan Synchrotron Radiation Research Institute (JASRI), Hyogo, Japan. Additionally, XPS was conducted using a Thermo Scientific Nexsa instrument, which was equipped with both an electron flood gun and a scanning ion gun. To study surface interactions, in situ DRIFTS experiments were performed on a Bruker FT‐IR Spectrometer. The system utilized a high‐temperature, high‐pressure DRIFTS reaction cell (Harrick Scientific Products Inc.), an MCT/A detector, and measurements were taken at a resolution of 4 cm^−1^. NH_3_‐TPSR measurements were conducted by a tube furnace (GSL‐1100X, Kejing) combined with a quadrupole mass spectrometer (MS, HPR‐20 EGA, Hiden).

More details about Material Characterisations can be found in the Supplementary Information.

### Catalytic Performance Evaluation

The catalytic NH_3_ decomposition performances were conducted in a fixed‐bed flow reactor connected with a mass flow controller (GF 125CXXC, Bronkhorst) to control the flow rate. In a typical experiment, 50 mg of the sieved catalyst sample (45 to 80 mesh) was fixed between the right and left quartz wools at the center of the quartz tube reactor (internal diameter of 4.5 mm), with a thermocouple placed in direct contact with the catalyst bed to monitor the real‐time temperature. The packed catalyst was heated to 700 °C at a ramp rate of 5 °C min^−1^ in a flow of 5% H_2_/Ar gas with a flow rate of 160 mL min^−1^, kept at 700 °C for 2.5 h to reduce the catalyst and then exposed to high‐purity NH_3_ gas (≥99.99%). The WHSV was adjusted to 30000 mL_NH3_ g_cat_^−1^ h^−1^ by controlling the flow rate. The reaction was then carried out at various temperatures, which increased from 450 to 650 °C in 50 °C increments, and steady‐state was allowed to reach before the product analysis by holding each temperature for 60 min. To determine the NH_3_ conversions, the MS equipped with a quadrupole analyzer and a secondary electron multiplier detector operating at 850 eV was used to analyze the composition of the product gas (N_2_, H_2_, and unreacted NH_3_). The accuracy of product analyses was further verified by back titration method, the experimental details of which have been reported in previous studies by our group.^[^ ^23^ ^]^ The NH_3_ conversion (X_NH3_) were calculated using:

(1) $$X_{NH_{3}}=\frac{{\left[NH_{3}\right]}_{\mathrm{inlet}}-{\left[NH_{3}\right]}_{\mathrm{outlet}}}{\left(1+{\left[NH_{3}\right]}_{\mathrm{outlet}}\right)\times {\left[NH_{3}\right]}_{\mathrm{inlet}}}\times 100\%$$

where [NH_3_]_inlet_ and [NH_3_]_outlet_ represent the concentrations of NH_3_ measured at the reactor inlet and outlet, respectively.^[^ ^5^ ^]^ The H_2_ production rate, with the unit of mmol H_2_ g_cat_^−1^ min^−1^, was then calculated based on X_NH3_ by:

(2) $$H_{2}\;\mathrm{production}\;\mathrm{rate}\;=\frac{\mathrm{WHSV}\times X_{NH_{3}}\times 1.5}{V_{m}\times 60}\;$$

where V_m_ is the molar volume of gas at standard conditions (24 mL mmol^−1^ at 25 °C and 1 bar).^[^ ^24^ ^]^

Kinetic studies were performed across various temperatures and WHSVs to verify that the reaction occurred within the kinetic regime, ensuring no limitations due to mass transfer. The Arrhenius plots and activation energies were calculated using data obtained under similar gas flow rates and compositions, but at different temperatures, while maintaining NH_3_ conversion below 30% to avoid equilibrium effects. As such, the activity measurements were conducted under steady‐state conditions controlled by reaction kinetics. All catalysts were tested twice to confirm the reproducibility of the results.

Reaction orders were determined by independently varying the concentrations of NH_3_, H_2_, or N_2_ while maintaining Ar as the balance gas. These experiments were performed at 350 °C with a total gas flow rate of 25 mL min^−1^ at atmospheric pressure. For NH_3_, its concentration was adjusted between 10% and 90%, with Ar as the balance gas. When determining the reaction order for H_2_, the concentration of NH_3_ was fixed at 20%, while H_2_ was varied between 10% and 80% with Ar as the balance gas. Similarly, for N_2_, the NH_3_ concentration remained constant at 20%, while N_2_ was adjusted within the same range and balanced by Ar.

### First‐principles Calculations

First‐principles calculations were carried out using the pseudopotential plane wave method based on DFT.^[^ ^25^ ^]^ These computations were performed with the Cambridge Serial Total Energy Package (CASTEP) software.^[^ ^26^ ^]^ The Perdew‐Burke‐Ernzerhof (PBE) functional within the generalized gradient approximation (GGA) framework was used to compute the exchange‐correlation energy.^[^ ^27^ ^]^ A cutoff energy of 400 eV was applied for the plane wave basis set.

The geometry of the Co(111)@BaAl_2_O_4_(202) interface was optimized using the Limited Memory Broyden‐Fletcher‐Goldfarb‐Shanno (LBFGS) algorithm.^[^ ^28^ ^]^ The convergence criteria for interatomic forces, internal stress, atomic displacements, and energy per atom between iterations were set to 0.05 eV Å^−1^, 0.1 GPa, 0.002 Å, and 2.0 × 10^−5^ eV atom^−1^, respectively. The Brillouin zone was sampled using a 2 × 5 × 1 Monkhorst‐Pack grid. Energy calculations were performed in reciprocal space to determine the total energy.

The *d*‐band centers for majority and minority spin states were calculated using the first moment of the density of states (DOS). The *d*‐band center was obtained using the Equation (3):^[^ ^29^ ^]^

(3) $$\varepsilon_{d\sigma }=\frac{\int_{-\infty }^{\infty }ED_{d\sigma }\left(E-E_{F}\right)dE}{\int_{-\infty }^{\infty }D_{d\sigma }\left(E-E_{F}\right)dE}\;$$

here, *D_dσ_* (*E*) represents the projected DOS of the transition metal's *d*‐states for spin *σ*, and *E_F_* is the Fermi energy of the system. Spin‐dependent fractional occupations were calculated using the following Equation (4):

(4) $$f_{\sigma }=\frac{\int_{-\infty }^{E_{F}}D_{d\sigma }\left(E\right)dE}{5}\;$$

### Statistical Analysis

The MS data was detected using the MASsoft 10 Professional. The SXRD data were treated using the program PDViPeR. The XANES data were background‐subtracted and normalized using the AUTOBK routine in Athena software. An established data reduction method was used to extract the EXAFS χ‐functions from the raw experimental data using the IFEFFIT software. The XPS data were processed by Computer aided surface analysis for X‐ray photoelectron spectroscopy (CasaXPS) software and the carbon 1s peak at 284.6 eV was used to calibrate all the spectra. The core particle size and shell thickness of catalysts were measured using TEM images with the help of Ganta DigitalMicrograph and Nano measurer softwares. All the figures were formatted in Origin. Details can be found in the “III. Material characterizations” of the Supporting Information.

## Conflict of Interest

The authors declare no conflict of interest.

## Supporting information



Supporting Information

## Data Availability

The data that support the findings of this study are available from the corresponding author upon reasonable request.
