# Supplementary material for: Efficient Low‐temperature Ammonia Cracking Enabled by Strained Heterostructure Interfaces on Ru‐free Catalyst
Source: Adv Mater. 2025 Apr 28;37(48):2502034. doi: 10.1002/adma.202502034 (PMC12676095; doi:10.1002/adma.202502034)
Supplement: Supplementary file 1 — Supporting Information [file ADMA-37-2502034-s001.docx]

Supporting Information

Efficient Low-temperature Ammonia Cracking Enabled by Strained Heterostructure Interfaces on Ru-free Catalyst

Pei Xiong, Jiangtong Li, Zhihang Xu, Yashan Lin, Robert David Bennett, Yi Zhang, Wei-Min Tu, Ye Zhu, Yun-Liang Soo, Tai-Sing Wu*‬‬‬‬‬‬‬‬‬‬‬‬‬‬‬‬‬‬‬‬‬‬‬‬‬‬‬‬‬‬‬‬‬‬‬‬‬‬‬‬‬‬‬‬‬‬‬‬‬‬‬‬‬‬‬‬‬‬‬‬‬‬‬‬‬‬‬‬‬‬‬‬‬‬‬‬‬‬‬‬‬‬‬‬‬‬‬‬‬‬‬‬‬‬‬‬‬‬‬‬‬‬‬‬‬‬‬‬‬‬‬‬‬‬‬‬‬‬‬‬‬‬‬‬‬‬‬‬‬‬‬‬‬‬‬‬‬‬‬‬‬‬‬‬‬‬‬‬‬‬‬‬‬‬‬‬‬‬‬‬‬‬‬‬‬‬‬‬‬‬‬‬‬‬‬‬‬‬‬‬‬‬‬‬‬‬‬‬‬‬‬‬‬‬‬‬‬‬‬‬‬‬‬‬‬‬‬‬‬‬‬‬‬‬‬‬‬‬‬‬‬‬‬‬‬‬‬‬‬‬‬‬‬‬‬‬‬‬‬‬‬‬‬‬‬‬‬‬‬‬‬‬‬‬‬‬‬‬‬‬‬‬‬‬‬‬‬‬‬‬‬‬‬‬‬‬‬‬‬‬‬‬‬‬‬‬‬‬‬‬‬‬‬‬‬‬‬‬‬‬‬‬‬‬‬‬‬‬‬‬‬‬‬‬‬‬‬‬‬‬‬‬‬‬‬‬‬, and

**This Supplementary Materials include:**

Supplementary Methods

Supplementary Figures 1 to 15

Supplementary Tables 1 to 4

# Supplementary Methods

## Materials and instruments

Iron nitrate nonahydrate (Fe(NO_3_)_3_·9H_2_O, 99.0%), cobalt nitrate hexahydrate (Co(NO_3_)_2_·6H_2_O, 99.0%), nickel nitrate hexahydrate (Ni(NO_3_)_2_·6H_2_O, 99.0%) and sodium carbonate (Na_2_CO_3_, 99.0%), sodium hydroxide (NaOH, 96.0%) and gamma-aluminium oxide (γ-Al_2_O_3_, 99.99% metals basis, ≤20nm) were purchased from Shanghai Aladdin Bio-Chem Technology Co., Ltd. Magnesium nitrate (Mg(NO_3_)_2_, 99.0%), calcium nitrate (Ca(NO_3_)_2_, 99.0%), barium nitrate (Ba(NO_3_)_2_, 99.0%) and aluminium nitrate nonahydrate (Al(NO_3_)_3_·9H_2_O, 99.0%) were obtained from Sigma-Aldrich. All chemicals with analytical-grade purity were used directly without further treatment unless otherwise noted. All solutions were prepared with deionized (DI) water. Anhydrous grade ammonia (NH_3_, ≥ 99.99%) and H_2_/Ar (5/95, v/v) were supplied by Scientific Gas Engineering Co., Ltd.

## Preparation of the M_T_-M_AE_-Al-O catalysts

The catalysts M_T_-M_AE_-Al-O (where M_T_ = Fe, Co, and Ni; M_AE_ = Mg, Ca, and Ba) with different compositions were synthesized using a co-precipitation method under automated pH control. In a typical synthesis, a 50 mL aqueous solution containing M_T_ (0.1 M), M_AE_ (0.05 M) and Al (0.1 M) at the desired molar ratio, 40M_T_:20M_AE_:40Al, was prepared by dissolving the corresponding metal nitrates in deionized (DI) water. At ambient temperature, the mixed-metal solution was gradually added dropwise into a stirred reactor (capacity: 500 mL to 2 L) containing 100 mL of 0.5 M Na_2_CO_3_ solution. The flow rate, controlled by a syringe pump, was set between 0.1 and 2.0 mL min^-1^. Vigorous stirring ensured uniform mixing throughout the process. Simultaneously, the pH of the reaction mixture was carefully regulated by the dropwise addition of a 4.0 M NaOH solution, also controlled by a syringe pump. Maintaining the pH was critical to selectively forming M_AE_CO_3_ instead of M_AE_(OH)_2_ precipitates. For this study, a pH value of 12.5 was used to produce BaCO_3_ and CaCO_3_, while a pH of 9.0 was chosen for MgCO_3_. After all solutions were added, the suspension was aged for 16 hours. Following aging, the mixture was filtered and rinsed with DI water until the filtrate reached a neutral pH (approximately 7.0). The resulting wet solid was re-dispersed in 200 mL of acetone and stirred for 2 hours at room temperature. The solid was then vacuum-filtered, thoroughly washed with acetone, and dried overnight in a vacuum oven at ambient conditions. The dried precipitate (100 mg) was subsequently calcined in air at 500 °C for 4 hours. Finally, the calcined sample underwent thermal treatment in a H_2_/Ar mixture (5/95, v/v) at 700 °C for 2.5 hours, yielding the M_T_-M_AE_-Al-O catalysts.

For the optimization of the Co-Ba-Al-O catalyst through adjustments to synthesis parameters, Co-Ba-Al-O catalysts were prepared using the above established preparation protocol while systematically varying synthesis factors. The Co content was adjusted across a range from 10.4 wt.% to 53.6 wt.%, and the Ba/Al ratios were modified from 0 to 3 to explore their influence on catalytic performance. Additionally, thermal treatments were applied under different calcination conditions, including no calcination, calcination at 500 °C , and calcination at 700 °C. Reduction temperatures were also varied, with treatments conducted at 600 °C , 700 °C, and 800 ^o^C. These systematic variations in synthesis parameters were designed to identify optimal conditions for enhancing the performance of the Co-Ba-Al-O catalyst.

## Material characterizations

### X-ray diffraction (XRD)

The crystalline phases and structural properties of the samples were determined using powder X-ray diffraction (XRD) analysis. The measurements were performed with a Rigaku SmartLab 9kW - Advanced diffractometer, which utilizes Cu Kα radiation (wavelength: 1.5406 Å). To prevent exposure of air-sensitive samples during XRD analysis, the materials were initially sealed inside a reaction tube fitted with closed ball valves and transferred to an inert gas glove box. Inside the glove box, the reaction tube was opened, and the samples were carefully placed in a quartz sample holder. The holder was then covered with Kapton film to maintain an airtight environment, ensuring that the samples remained protected from air during the XRD measurements.

The crystallite size (D) of the samples was calculated using the Debye-Scherrer equation, which connects the size of crystallites to the broadening of XRD peaks. The equation is given as:

$$\begin{aligned} D=\frac{k\lambda}{\beta cos\theta}\#\left( 1 \right) \end{aligned}$$

Here, D represents the size of the crystallite, k is a shape factor dependent on the crystal geometry and reciprocal lattice point (commonly taken as 0.9), *λ* is the wavelength of the X-ray source (Cu Kα), *β* is the full width at half-maximum (FWHM) of the diffraction peak, and θ is the Bragg angle.

### Synchrotron X-ray diffraction (SXRD)

Synchrotron X-ray diffraction (SXRD) patterns were obtained at the Powder Diffraction (PD) beamline of the Australian Synchrotron (AS), with the photon energy set at 17.711 keV (corresponding to a wavelength of λ = 0.7000 Å). The samples were prepared in capillaries, which were mounted on a goniometer head and rotated at a speed of 15 rpm during measurement. The diffraction patterns were recorded using Debye-Scherrer geometry, with a goniometer diameter of 152 cm, covering a 2θ range from 1.01398° to 80.8344° and an effective step size of 0.00375°. Both the incident and diffracted X-ray beams were fully polarized.

For each sample, two sets of data were collected with acquisition times of 450 seconds per measurement. The Mythen detector was offset by 0.5° for the second measurement to allow better data merging. The two offset patterns were combined using the PDViPeR software. Additionally, to calibrate the instrument parameters, such as the wavelength, 2θ_0_ correction, and full width at half maximum (FWHM) of the Bragg peaks, a reference pattern was recorded for NIST SRM-660b LaB_6_ powder under identical experimental conditions.

### In situ synchrotron X-ray diffraction (In situ SXRD)

In situ SXRD was employed to study the structural transformations of NH_3_-adsorbed Co@BaAl_2_O_4-x_ during the desorption process. The SXRD patterns of Co@BaAl_2_O_4-x_-NH_3_, with pre-adsorbed NH_3_, were recorded as the temperature increased from -173 °C to 227 °C. These measurements were performed at the Powder Diffraction (PD) beamline of the Australian Synchrotron (AS), with the photon energy set at 17.711 keV (wavelength λ = 0.7000 Å). The preparation of the NH_3_-adsorbed sample involved placing 50 mg of Co@BaAl_2_O_4-x_ in a quartz tube under vacuum conditions. The sample was heated to 300 °C to remove any residual gases or contaminants. Following this degassing step, a controlled amount of NH_3_ gas was introduced into the vacuum chamber containing the sample. NH_3_ was allowed to adsorb onto the sample surface for 30 minutes. To preserve the adsorbed NH_3_, the sample was carefully transferred into a capillary tube inside a glovebox, ensuring an inert environment.

During the SXRD measurements, temperature control was achieved using Oxford Cryostream equipment.^[1]^ The temperature was gradually increased from 100 K to 480 K at a ramp rate of 6 K/min. To ensure thermal stability, the system was held at each target temperature (-173, -73, 27, 127, and 227 °C) for 20 minutes. SXRD patterns were collected at the end of the dwell period to capture the structural changes occurring at each temperature during the NH_3_ desorption process.

### Transmission electron microscopy (TEM)

The microstructure and morphology of the catalysts were characterized by using scanning electron microscopy (SEM) and transmission electron microscopy (TEM) imaging. High-angle annular dark-field (HAADF) STEM images and Electron energy loss spectroscopy in scanning transmission electron Microscopy (EELS-STEM) were acquired using the JEOL JEM-2100F microscope (Japan) operated at 200 kV equipped with a Gatan Enfina electron spectrometer (USA). Spherical· aberration-corrected scanning transmission electron microscopy (AC-STEM) was conducted using double-Cs-corrected STEM (Spectra 300, TFS, USA). To prepare TEM specimens, a small amount of material was dispersed in 1 mL of ethanol and sonicated for 180 seconds. The resulting suspension was then deposited dropwise onto copper grids. For samples that were either reduced or subjected to reactions, additional precautions were taken to prevent exposure to air. These samples were sealed in a reaction tube with closed ball valves and transferred directly into an inert gas glove box. Inside the glove box, the samples were removed from the reaction tube, dispersed in ethanol, and subsequently deposited onto copper grids for TEM analysis.

### X-ray absorption fine structure (XAFS)

XAFS measurements were conducted in fluorescence mode using a Lytle detector at beamline BL01C of the Taiwan Light Source, operated by the National Synchrotron Radiation Research Center (NSRRC), Hsinchu, Taiwan. Photon energy was scanned using a Si(111) double-crystal monochromator (DCM). To ensure the reliability of the experimental results, each sample was measured at least twice, and the scans were compared to confirm reproducibility.

X-ray absorption near-edge structure (XANES) data were processed by subtracting the background and normalizing the spectra using the AUTOBK routine in Athena software. Information regarding the radial distribution of atoms surrounding Co and Ba was obtained by analyzing the extended X-ray absorption fine structure (EXAFS). The raw experimental data were reduced to extract the EXAFS χ-functions using the IFEFFIT software package, following a well-established protocol. Wavelet transforms of the EXAFS spectra were performed using k^3^-weighted data. The Morlet wavelet mother function was applied across a k-range of 3 to 13 Å^-1^ for the Co K-edge. This choice of wavelet was driven by its ability to isolate the rapidly oscillating portion of the data within a Gaussian envelope, with its real and imaginary components closely resembling the features of an EXAFS spectrum.

### In situ X-ray absorption fine structure (In situ XAFS)

The oxidation state and local atomic environment around Co atoms during NH_3_ desorption/decomposition were investigated using in situ XAFS at the Co K-edge. The analysis was carried out under controlled heating conditions with the sample enclosed in a capillary. These measurements were performed in fluorescence mode using a 19SSD detector at the beamline BL01B1 of the SPring-8 (Japan Synchrotron Radiation Research Institute, Hyogo, Japan). The in situ XAFS setup included a quartz tube, a sample holder (or boat), a heating unit, and a temperature controller. The samples were heated from room temperature to 200 °C at a ramp rate of 10 °C min^-1^, and data were collected continuously during the temperature increase. The XAS spectra were processed and analyzed using the Athena software, which is part of the IFEFFIT package.

### X-ray photoelectron spectroscopy (XPS)

X-ray photoelectron spectroscopy (XPS) experiments targeting O 1s, Co 3p, Ba 4d, and Al 2p were conducted using a Thermo Scientific Nexsa instrument equipped with a scanning ion gun and an electron flood source. The binding energy (BE) calibration was performed using the adventitious carbon C 1s peak at 284.8 eV as a standard reference. The Shirley background was subtracted before deconvoluting the XPS spectra, which was performed using Gaussian-Lorentzian fitting functions. The surface chemical composition of the samples was determined by analyzing the integrated areas of the deconvoluted spectral peaks. To measure the post-reaction samples, they were sealed within the reaction tube using closed ball valves at both ends and directly transferred into an inert gas glove box. In the inert gas glove box, the samples were taken from the reaction tube and loaded into a vacuum transfer module, which was then evacuated to a high vacuum using an integrated pump and transported into the loading chamber of the XPS system. This transfer method ensured that the chemical composition of the sample surface was not compromised through the prevention of unwanted surface oxidation reactions.

### In situ diffuse reflectance infrared Fourier transform spectroscopy (In situ DRIFTS)

In situ DRIFTS experiments were conducted using a Bruker Fourier Transform Infrared (FT-IR) spectrometer equipped with a high-temperature, high-pressure DRIFTS reaction cell (Harrick Scientific Products Inc.). The measurements utilized an MCT/A detector with a spectral resolution of 4 cm^-1^. For the NH_3_ decomposition study, 50 mg of the sample was pre-treated under a pure nitrogen (N_2_) flow (10 mL min^-1^) at 400 °C. The activation process involved heating the sample to 400 °C over 2 hours, followed by maintaining this temperature for an additional 4 hours. Once the activation step was complete, the system was cooled to room temperature, and the sample was scanned using a KBr background spectrum under an N_2_ atmosphere. After recording the background spectrum, a flow of 10% NH_3_ (20 mL min^-1^) was introduced for 20 minutes. Following this, the NH_3_ flow was discontinued, and the inlets an d outlets of the sample holder were sealed to isolate the system.

Spectra were recorded at room temperature as well as at 100 °C, 200 °C, 300 °C, and 400 °C, with the background spectrum collected under N_2_ at room temperature being used for reference. Additional scans were performed after the temperature reached 400 °C, at time intervals of 0, 15, 30, 45, 60, and 120 minutes. Each scan consisted of 64 accumulations to ensure high-quality spectral data.

### NH_3_ temperature-programmed surface reaction (NH_3_-TPSR)

NH_3_ temperature-programmed surface reaction (NH_3_-TPSR) measurements of samples were conducted using a tube furnace (GSL-1100X, Kejing) combined with a quadrupole mass spectrometer (HPR-20 EGA, Hiden). Inside the quartz tube (Inner diameter: 4.5 mm, Outer diameter: 6.0 mm), 50 mg of sample, sieved to a particle size range of 45 to 80 mesh, was sandwiched between two layers of quartz wool with a thermocouple in contact with the sample. To ensure NH_3_ chemisorption on the Co surface, NH_3_ gas was introduced into the reactor at 50 °C for 60 minutes prior to the collection of NH_3_-TPSR signals. Following this chemisorption step, the sample was heated from 50 to 800 °C with the purge of Ar. The signals of H_2_ (m/z = 2), NH_3_ (m/z = 17), and N_2_ (m/z = 28) were tracked during the investigation.


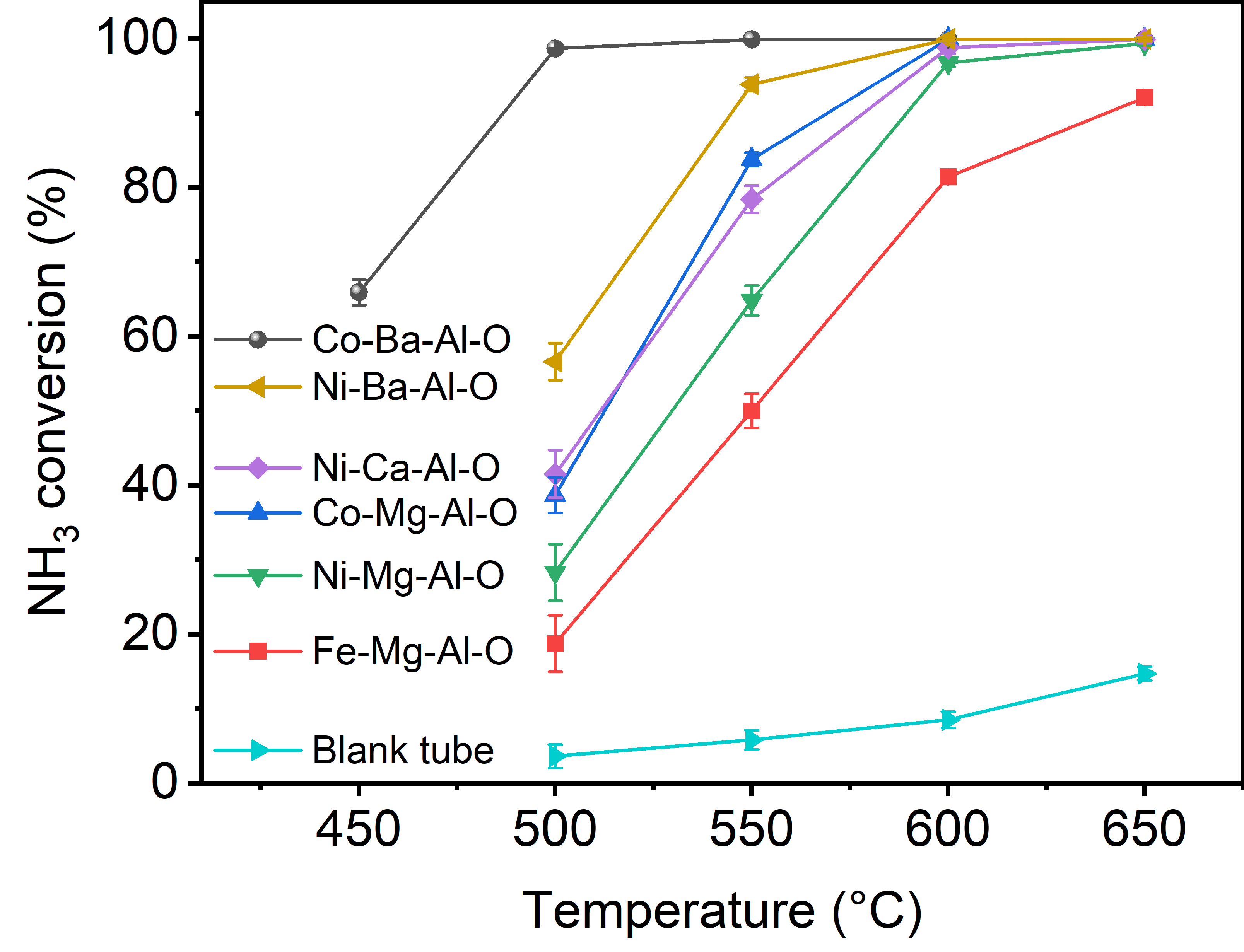


**Figure S1.** NH_3_ decomposition conversion over catalysts with different active metals and promoters. Among these, Co-Ba-Al-O composition exhibits superior activity for the low temperature (< 600 °C) range. The performance was evaluated at WHSV of 30,000 mL g_cat_^-1^ h^-1^ and 1 bar.


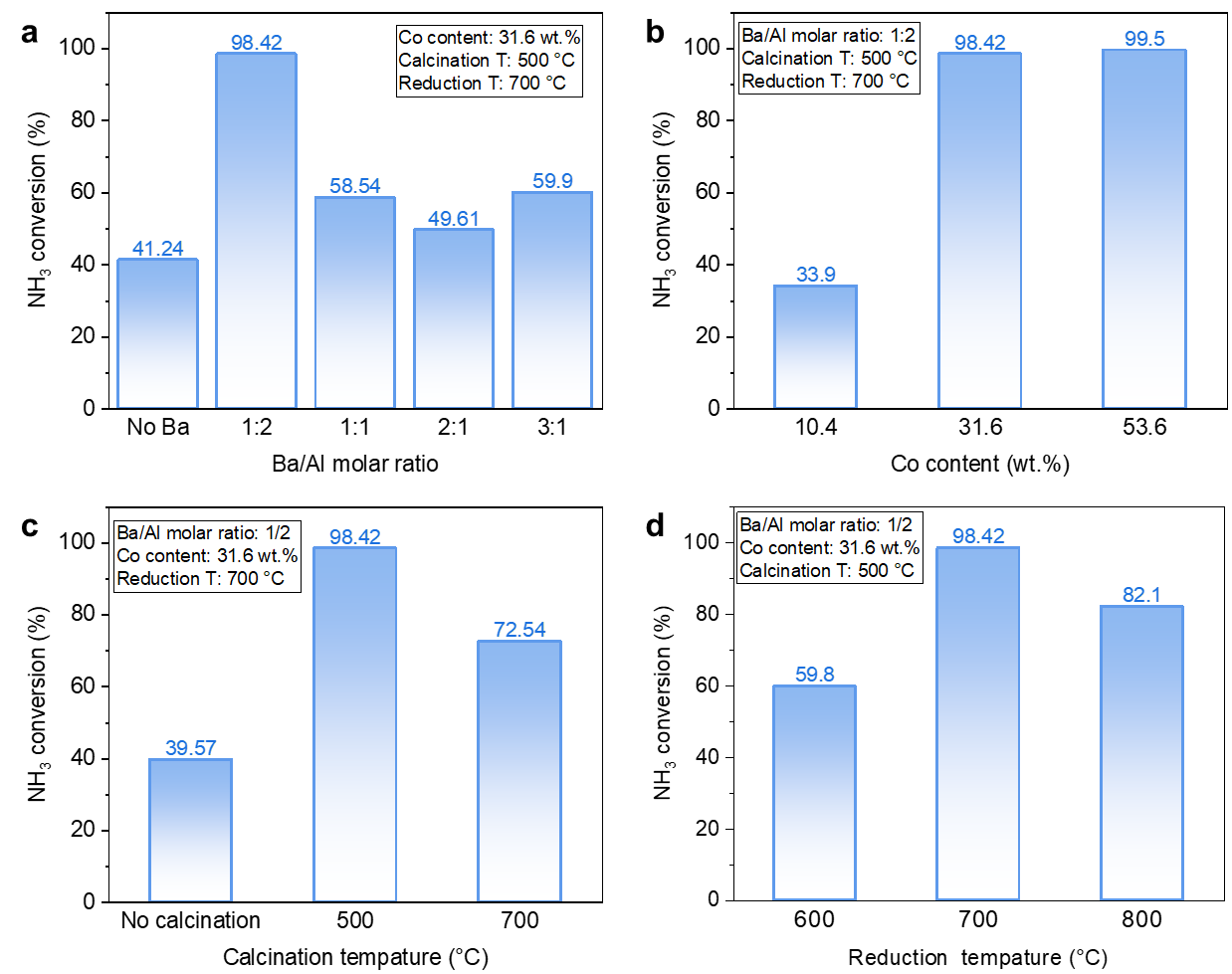


**Figure S2.** Refinement of the Co-Ba-Al-O catalyst by adjusting synthesis parameters: a) NH_3_ conversion as a function of Ba/Al molar ratio over Co-Ba-Al-O while maintaining Co content (31.6 wt.%), calcination temperature (500 °C), and reduction temperature (700 °C). b) NH_3_ conversion as a function of Co content over Co-Ba-Al-O while maintaining Ba/Al molar ratio (1:2), calcination temperature (500 °C), and reduction temperature (700 °C). c) NH_3_ conversion as a function of calcination temperature over Co-Ba-Al-O while maintaining Ba/Al molar ratio (1:2), Co content (31.6 wt.%), and reduction temperature (700 °C). d) NH_3_ conversion as a function of reduction temperature over Co-Ba-Al-O while maintaining Ba/Al molar ratio (1:2), Co content (31.6 wt.%), and calcination temperature (500 °C). Reaction conditions: WHSV = 30,000 mL g_cat_^-1^ h^-1^, 1 bar.


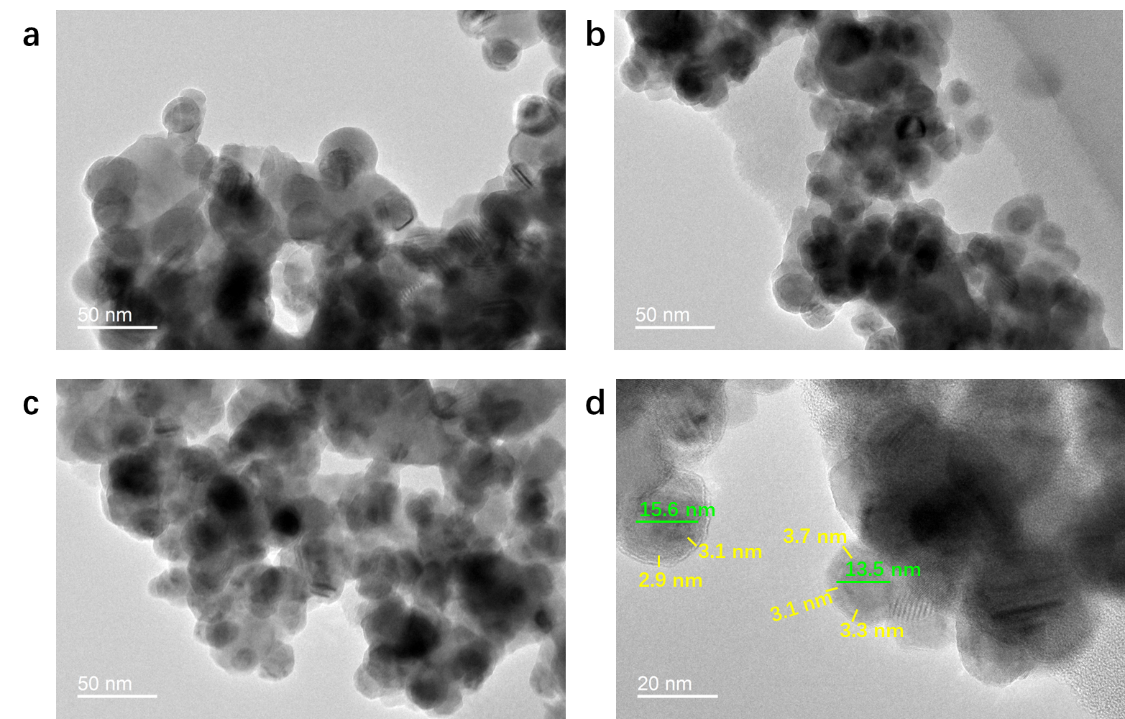


**Figure S3.** a)-c) Low-magnification transmission electron microscopy (TEM) image of Co@BaAl_2_O_4-x_ heterostructure. d) Illustration of the measurement of the shell thickness (yellow lines) and core particle size (green lines) of core@shell particles.

The core size and shell thickness of core@shell particles were manually measured using TEM images. For core size determination, a straight line was drawn across the widest axis of each core particle, and the measurement was converted into physical dimensions using the scale bar provided in the images. To analyze shell thickness, radial lines were extended outward from the center of the core through the shell region. The thickness was calculated by subtracting the core radius from the combined core+shell radius, based on the corresponding scale bar. Measurements were conducted at multiple positions along the shell of each particle to account for potential variations in thickness. To ensure statistical robustness, at least 50 individual core@shell particles were analyzed. The compiled measurements were used to construct a size distribution chart for both core size and shell thickness. A Gaussian fitting was applied to describe the overall distribution, providing insights into the uniformity of these dimensions across the sample.


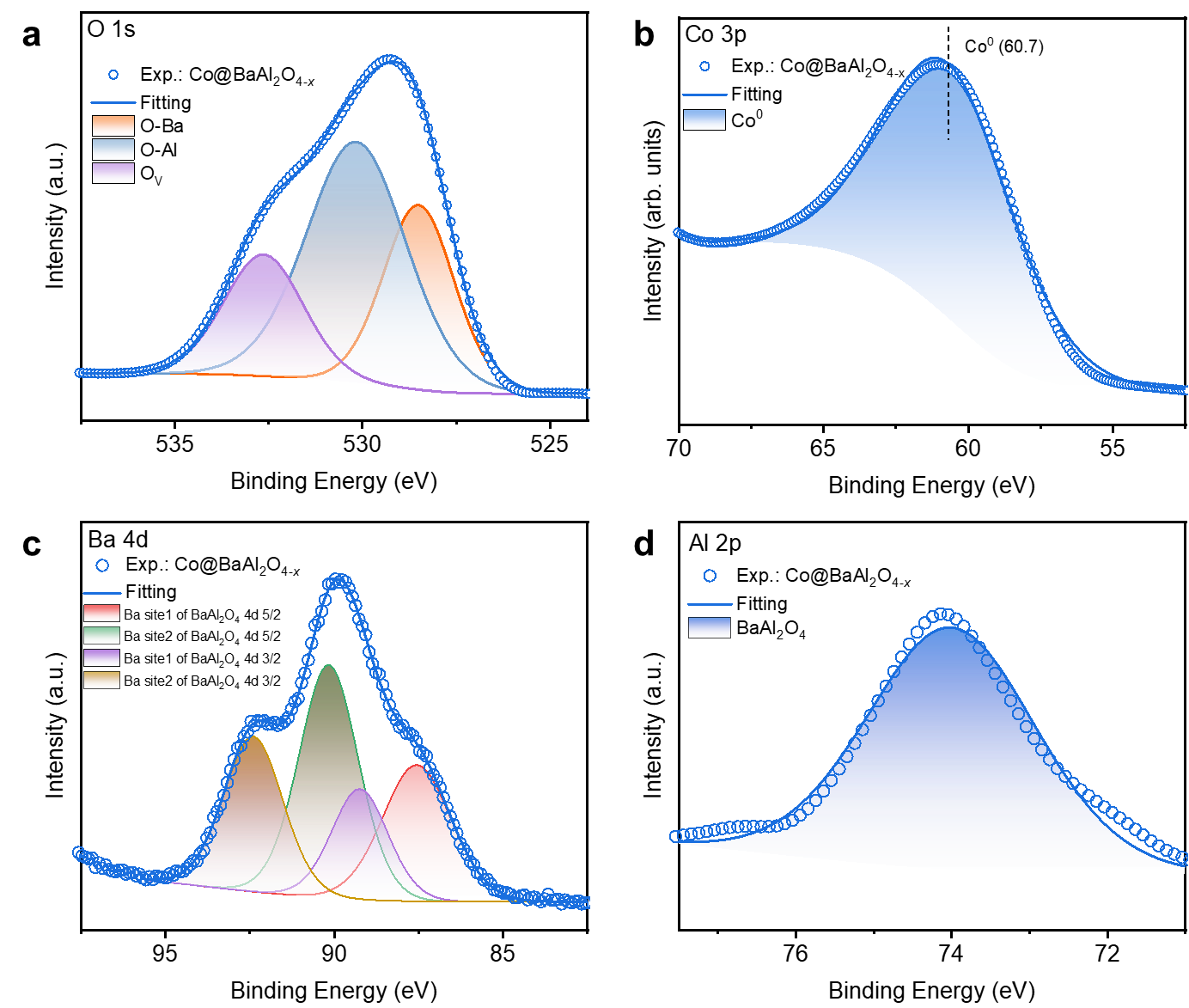


**Figure S4.** a) O 1s X-ray photoelectron spectroscopy (XPS) of Co@BaAl_2_O_4-x_. b) Co 3p X-ray photoelectron spectroscopy (XPS) of Co@BaAl_2_O_4-x_. c) Ba 4d X-ray photoelectron spectroscopy (XPS) of Co@BaAl_2_O_4-x_. d) Al 2p X-ray photoelectron spectroscopy (XPS) of Co@BaAl_2_O_4-x_.


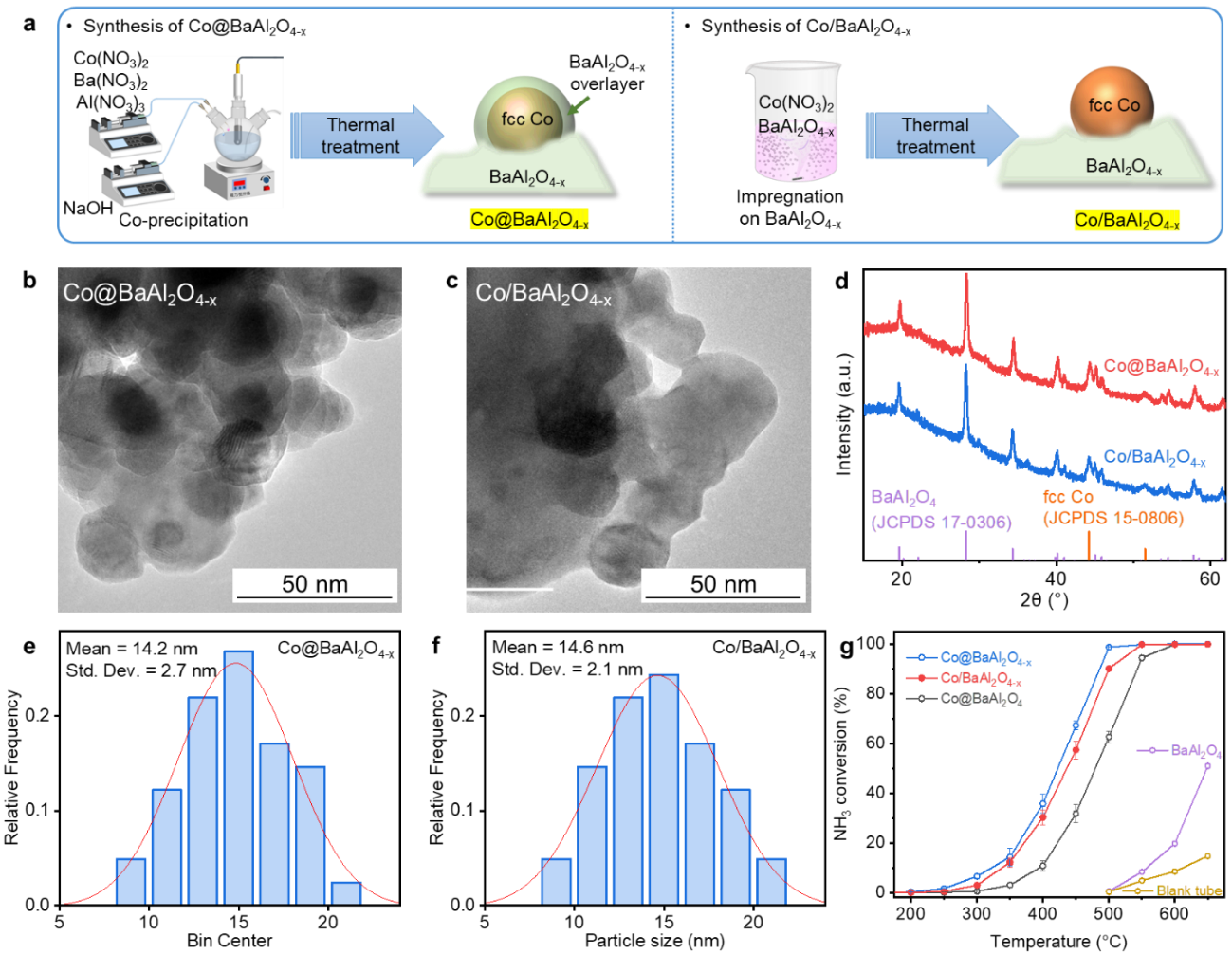


**Figure S5.** a) Schematic illustration of synthesis details for Co@BaAl_2_O_4-x_ and Co/BaAl_2_O_4-x_. b) Transmission electron microscopy (TEM) images of Co@BaAl_2_O_4-x_. c) Transmission electron microscopy (TEM) images of Co/BaAl_2_O_4-x_. d) Laboratory X-ray diffraction (XRD) patterns of Co@BaAl_2_O_4-x_ and Co/BaAl_2_O_4-x_. e) Size distributions of Co NPs measured from low magnification TEM images of Co@BaAl_2_O_4-x_. f) Size distributions of Co NPs measured from low magnification TEM images of Co/BaAl_2_O_4-x_. g) NH_3_ conversion as a function of reaction temperature (200 to 650 °C) over Co@BaAl_2_O_4-x_, Co/BaAl_2_O_4-x_, and the Co@BaAl_2_O_4_ sample treated to remove oxygen vacancy (denoted as Co@BaAl_2_O_4_, see thermal treatment process in Figure S6). Reaction conditions: WHSV = 30,000 mL g_cat_^-1^ h^-1^, 1 bar.


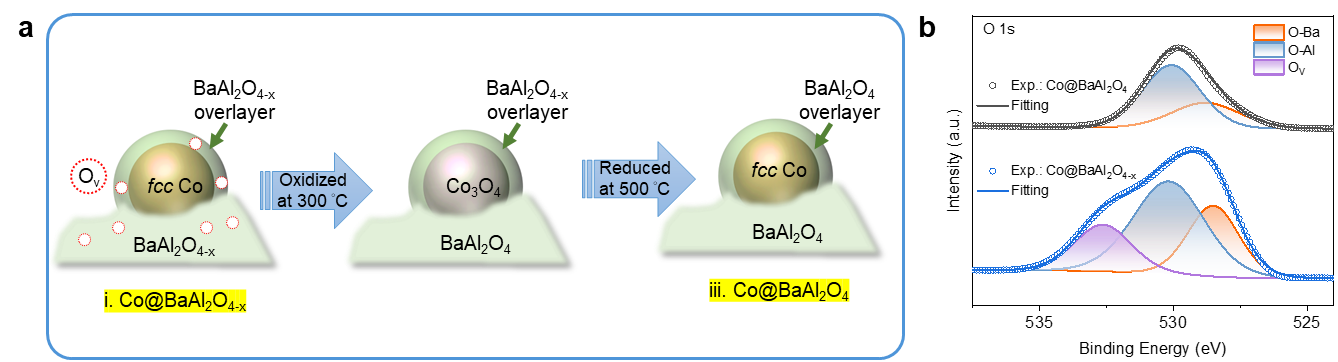


**Figure S6.** a) Schematic illustration of thermal treatment details for obtaining the Co@BaAl_2_O_4_ catalyst. Co@BaAl_2_O_4-x_ was oxidized in pure oxygen at 300 °C to reduce oxygen vacancies (O_v_). Subsequently, the samples were treated under H_2_/Ar at 500 °C to reduce the reoxidized Co_3_O_4_ to Co NPs, resulting in the formation of defect-free samples denoted as Co@BaAl_2_O_4_. b) O 1s XPS of Co@BaAl_2_O_4-x_ and Co@BaAl_2_O_4_.

**Table S1.** Lattice parameters of Co@BaAl_2_O_4-x_ and Co/BaAl_2_O_4-x_ determined by Rietveld analysis.

|  | BaAl_2_O_4_, P63 (173), hcp, JCPDS71-1323 | | | Co, Fm-3m (225), fcc, JCPDS15-0806 | | |
| --- | --- | --- | --- | --- | --- | --- |
|  | Lattice Parameter, Å | Cell volume, Å^3^ | Crystallite size, nm | Lattice parameter, Å | Cell volume, Å^3^ | Crystallite size, nm |
| Co@BaAl_2_O_4-x_ | a = b = 10.49140  c = 8.84774 | 5060.20819 | 48.8 ± 2.5 | a = b = c = 3.57613 | 45.73407 | 18.8 ± 3.4 |
| Co/BaAl_2_O_4-x_ | a = b = 10.50387  c = 8.82240 | 5057.71744 | 63.4 ± 1.5 | a = b = c = 3.57596 | 45.72755 | 13.2 ± 3.9 |

**Figure S7.** Dependence of NH_3_ decomposition rate of Co@BaAl_2_O_4-x_ and Co/BaAl_2_O_4-x_ on the partial pressures of N_2_ at 350 °C.

**
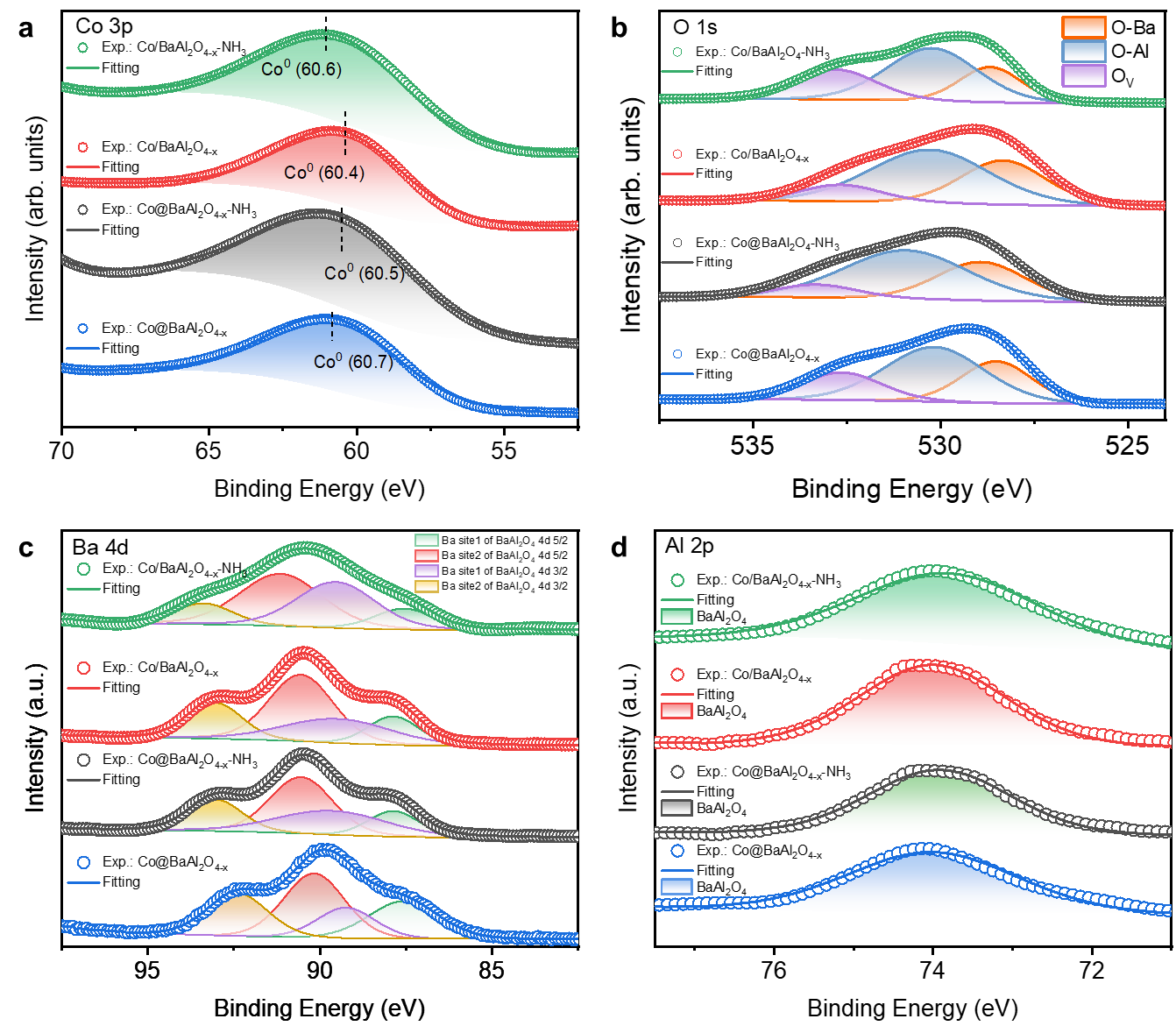
**

**Figure S8.** a) Co 3p, b) O 1s, c) Ba 4d, and d) Al 2p X-ray photoelectron spectroscopy (XPS) of Co@BaAl_2_O_4-x_, Co@BaAl_2_O_4-x_-NH_3_, Co/BaAl_2_O_4-x_, and Co/BaAl_2_O_4-x_-NH_3_.

**
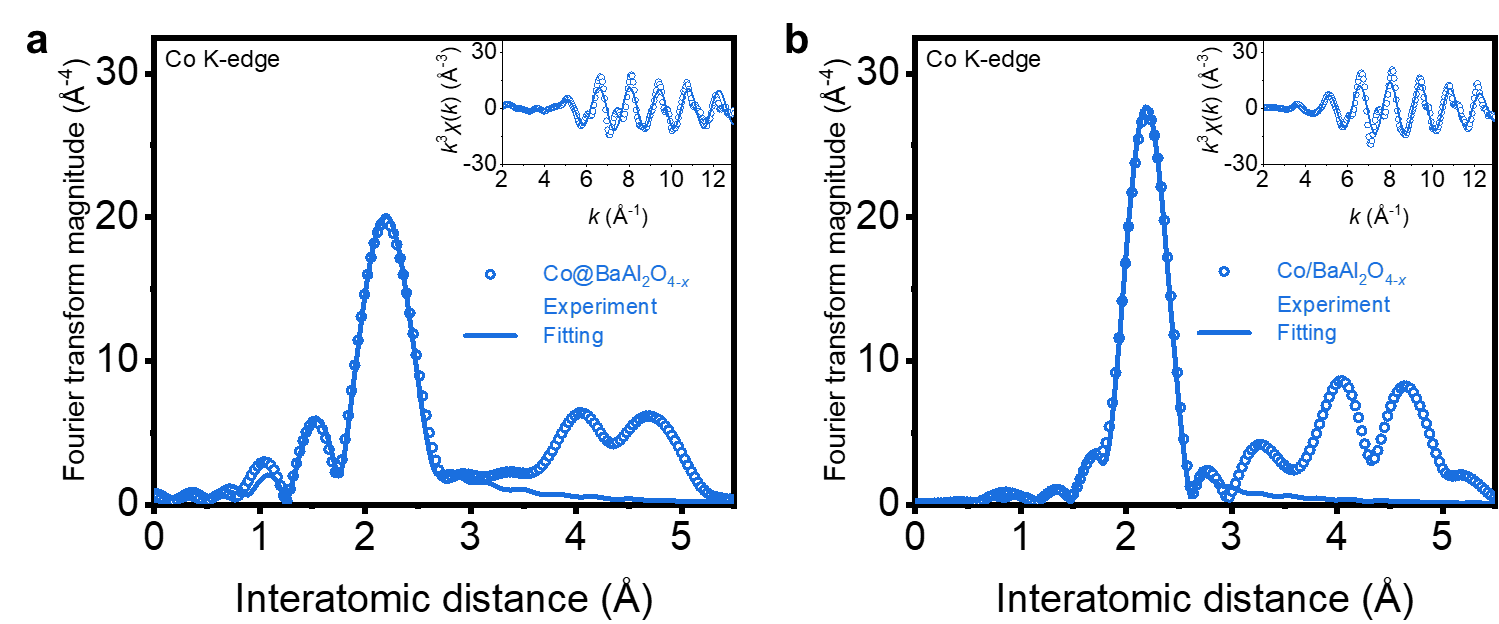
**

**Figure S9.** a) Co K-edge EXAFS fitting curves of Co@BaAl_2_O_4-x_. b) Co K-edge EXAFS fitting curves of Co/BaAl_2_O_4-x_.

**Supplementary Table S2.** Co K-edge EXAFS fitting parameters of Co@BaAl_2_O_4-x_, and Co/BaAl_2_O_4-x_.

| Sample | Bond | CN | R, Å | D-W factor (σ^2^), Å^2^ | ΔE_0_, eV | R-factor, % |
| --- | --- | --- | --- | --- | --- | --- |
| Co@BaAl_2_O_4-x_ | Co-O | 1.5 (2) | 1.96 (2) | 0.003 (2) | 9.7 (5) | 1.0 |
|  | Co-Co | 3.5 (1) | 2.49 (1) | 0.003 (1) | 8.3 (4) |  |
|  | Co-Co | 0.5 (2) | 2.50 (1) | 0.003 (2) |  |  |
| Co/BaAl_2_O_4-x_ | Co-Co | 6.3 (3) | 2.49 (1) | 0.004 (1) | 7.5 (5) | 0.3 |
| Co foil | Co-Co | 12.0 | 2.49 (1) | 0.006 (1) | 7.1 (4) | 0.2 |

*Co K-edge EXAFS fitting results, in which CN is the average coordination number, R is the distance from the absorber atom, and σ^2^ is the Debye–Waller factor. R-factor denotes the quality factor of the fitting, and ΔE_0_ the energy shift from the absorption edge energy E_0_.

**Fitting parameters: k = 3.0 – 13.6; R = 1.0 – 3.0; amp = 0.79.


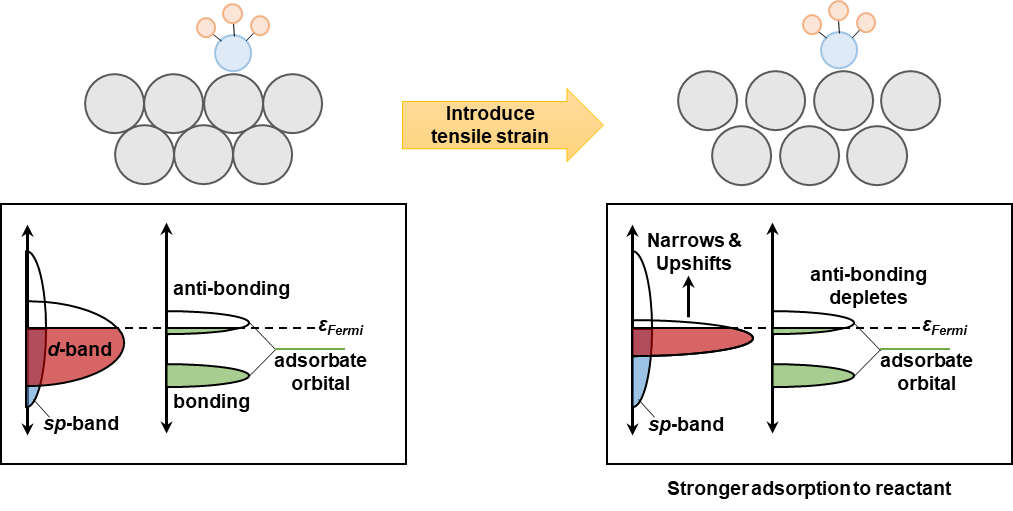


**Figure S10.** Schematic diagrams explaining the effect of tensile strain on the *d*-band position of metals.^[2]^

## Supplementary Information for First-principles calculations

The SXRD (Figure 3a,b), XAS (Figure 4b), and STEM (Figure 3c-d) results in the main manuscript confirm the lattice strain induced by the core@shell heterostructure at the interface. To enhance the accuracy of the DFT modeling, we utilized XRD results from Figure 3a and Figure S5d, showing NH_3_ adsorption primarily on the Co(111) surface, guiding the creation of a Co(111) surface model. Drawing from our prior work characterizing similar catalysts,^[3]^ where the shell material is a BaAl_2_O_4_ with a P6_3_22 space group, we obtained the corresponding structure from the Materials Project Database to combine with the Co(111) surface for heterostructure construction.

As illustrated in Figure S11a-b, the bond length at the strained-Co(111) heterostructure interface approximates 2.488 Å, longer than that on the unstrained-Co(111) surface (2.478 Å). This DFT calculation result demonstrates that tensile strain occurs at the heterostructure interface, likely due to a lattice mismatch between the Co and BaAl_2_O_4_ heterostructure interface.

Additionally, the electronic changes observed were further substantiated through X-ray absorption near edge structure (XANES) simulations executed using the finite difference method near edge structure (FDMNES) code on the strained-Co(111) heterostructure and unstrained-Co(111) models. Consistent with the experimental findings from XANES in Figure 4a, it is discerned that the strained-Co(111) heterostructure displays a higher valence in comparison to the unstrained-Co(111) surface, thereby validating that the heterostructure-induced tensile lattice strain augments the Co valence.


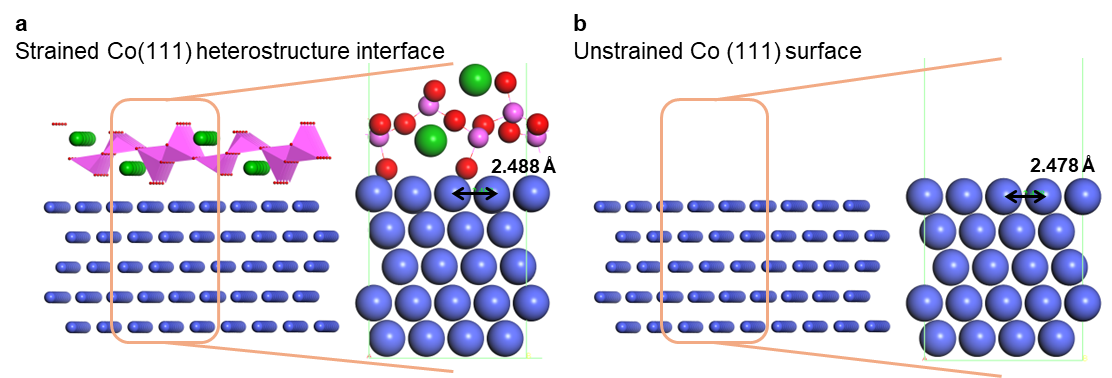


**Figure S11.** a) Optimized strained Co(111) heterostructure interface for density functional theory (DFT) calculations. b) Optimized unstrained Co(111) surface for density functional theory (DFT) calculations.

**Figure S12.** Co K-edge XANES simulation results of strained Co(111) heterostructure interface and unstrained Co(111) surface models using finite difference method near edge structure (FDMNES) code.^[4]^


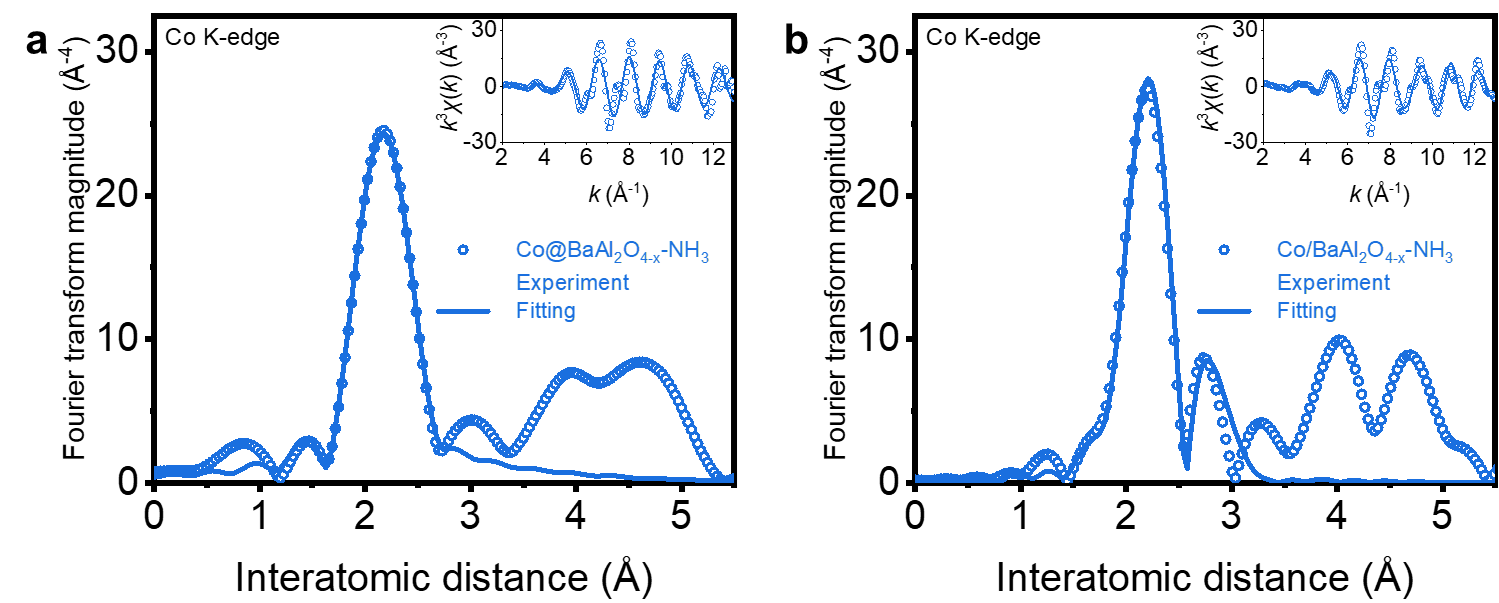


**Figure S13.** a) Co K-edge EXAFS fitting curves of Co@BaAl_2_O_4-x_-NH_3_. b) Co K-edge EXAFS fitting curves of Co/BaAl_2_O_4-x_-NH_3_.

**Table S3.** Co K-edge EXAFS fitting parameters of Co@BaAl_2_O_4-x_-NH_3_, and Co/BaAl_2_O_4-x_-NH_3_.

| Sample | Bond | CN | R, Å | D-W factor (σ^2^), Å^2^ | ΔE_0_, eV | R-factor, % |
| --- | --- | --- | --- | --- | --- | --- |
| Co@BaAl_2_O_4-x_-NH_3_ | Co-N (NH_3_) | 0.5 (2) | 1.76 (2) | 0.004 (3) | 6.8 (7) | 0.5 |
|  | Co-O | 1.3 (4) | 1.92 (2) | 0.003 (3) | -3.5 (5) |  |
|  | Co-Co | 7.2 (2) | 2.49 (1) | 0.004 (1) | 7.1 (3) |  |
| Co/BaAl_2_O_4-x_-NH_3_ | Co-N (NH_3_) | 0.2 (1) | 1.76 (3) | 0.013 (4) | 9.9 (8) | 0.8 |
|  | Co-Co | 6.0 (6) | 2.49 (1) | 0.004 (1) | 7.6 (4) |  |
|  | Co-N (CoN_3_) | 1.2 (2) | 2.09 (3) | 0.003 (2) | 9.9 (8) |  |
|  | Co-N (CoN_3_) | 1.8 (5) | 2.69 (2) | 0.014 (7) |  |  |
|  | Co-Co (CoN_3_) | 3.8 (4) | 3.26 (2) | 0.004 (1) | 7.6 (4) |  |
| Co foil | Co-Co | 12.0 | 2.49 (1) | 0.006 (1) | 7.1 (4) | 0.2 |

*Co K-edge EXAFS fitting results, in which CN is the average coordination number, R is the distance from the absorber atom, and σ^2^ is the Debye–Waller factor. R-factor denotes the quality factor of the fitting, and ΔE_0_ the energy shift from the absorption edge energy E_0_.

**Fitting parameters: k = 3.0 – 13.6; R = 1.0 – 3.0; amp = 0.79.

**Figure S14.** In situ synchrotron X-ray diffraction (SXRD) full patterns of Co@BaAl_2_O_4-x_-NH_3_ and Co/BaAl_2_O_4-x_-NH_3_ with temperature raising from -173 °C to 227 °C.


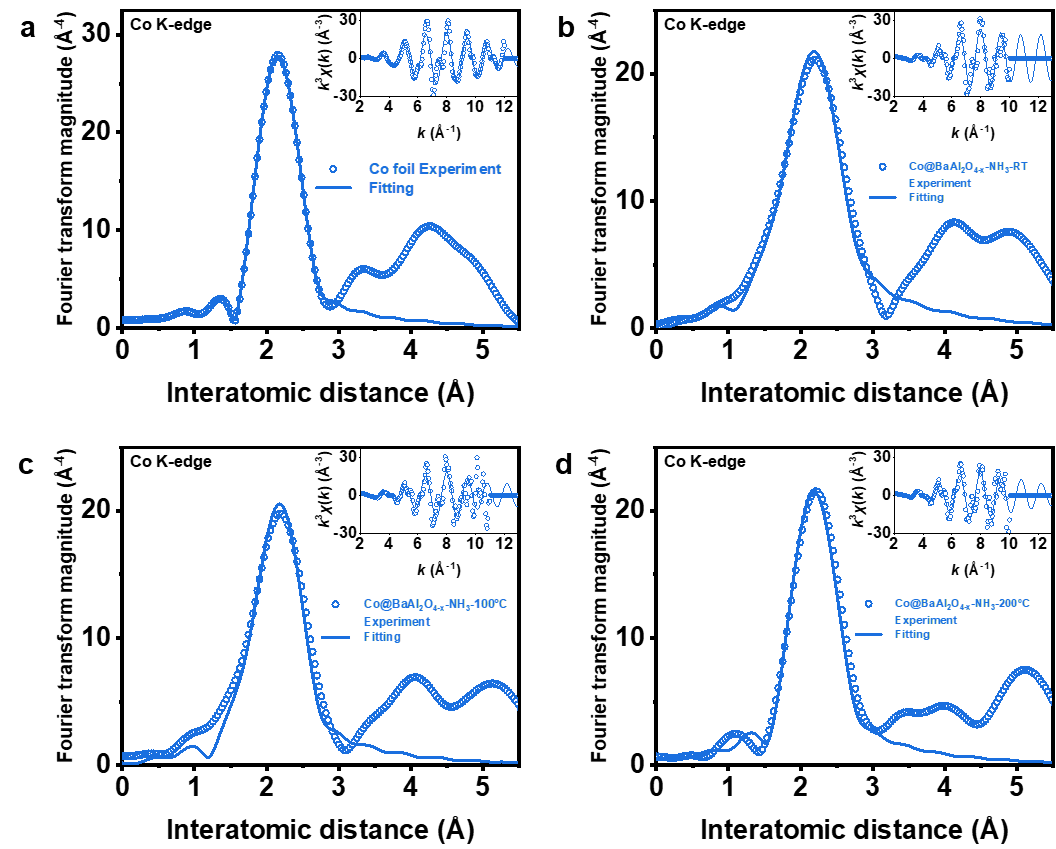


**Figure S15.** In situ Co K-edge EXAFS fitting curves of Co@BaAl_2_O_4-x_-NH_3_ under heating at different temperatures: a) Co foil; b) room temperature; c) 100 °C; d) 200 °C.

**Table S4.** Co K-edge EXAFS fitting parameters of Co@BaAl_2_O_4-x_-NH_3_ under heating at different temperatures.

| Sample | Bond | CN | R, Å | D-W factor (σ^2^), Å^2^ | ΔE_0_, eV | R-factor, % |
| --- | --- | --- | --- | --- | --- | --- |
| Co@BaAl_2_O_4-x_-NH_3_-RT | Co-N (NH_3_) | 2.1 (7) | 2.00 (1) | 0.003 (3) | -6.0 (8) | 1.8% |
|  | Co-Co | 7.7 (4) | 2.51 (1) | 0.003 (1) | 9.9 (6) |  |
| Co@BaAl_2_O_4-x_-NH_3_-100°C | Co-N (NH_3_) | 2.3 (4) | 2.03 (3) | 0.005 (1) | -2.3 (5) | 1.8% |
|  | Co-Co | 7.3 (5) | 2.50 (1) | 0.005 (1) | 9.9 (6) |  |
|  | Co-Co | 1.0 (3) | 2.55 (2) | 0.010 (3) |  |  |
| Co@BaAl_2_O_4-x_-NH_3_-200°C | Co-Co | 6.8 (7) | 2.50 (1) | 0.006 (2) | 9.9 (8) | 1.9% |
|  | Co-Co | 2.9 (5) | 2.56 (2) | 0.012 (5) |  |  |
| Co foil | Co-Co | 12.0 | 2.49 (1) | 0.006 (1) | 6.9 (4) | 0.1 |

*Co K-edge EXAFS fitting results, in which CN is the average coordination number, R is the distance from the absorber atom, and σ^2^ is the Debye–Waller factor. R-factor denotes the quality factor of the fitting, and ΔE_0_ the energy shift from the absorption edge energy E_0_.

**Fitting parameters: k = 3.0 – 13.6; R = 1.0 – 3.0; amp = 0.79.

# References

[1] D. Cluster, Sample environments of Australian Synchrotron PD beamline, **2025**, <https://asuserwiki.atlassian.net/wiki/spaces/UO/pages/392069366/Sample+environments>

[2] Q. Hu, K. Gao, X. Wang, H. Zheng, J. Cao, L. Mi, Q. Huo, H. Yang, J. Liu, C. He, Subnanometric Ru clusters with upshifted D band center improve performance for alkaline hydrogen evolution reaction, *Nat. Commun.* **2022**, 13, 3958.

[3] P. Xiong, Z. Xu, T. S. Wu, T. Yang, Q. Lei, J. Li, G. Li, M. Yang, Y. L. Soo, R. D. Bennett, S. P. Lau, S. C. E. Tsang, Y. Zhu, M. M. Li, Synthesis of core@shell catalysts guided by Tammann temperature, *Nat. Commun.* **2024**, 15, 420.

[4] O. Bunău, A. Y. Ramos and Y. Joly, *International Tables for Crystallography*, Springer Netherlands, Dordrecht, **2021**.
